# Supplementary material for: Expansion microscopy reveals nano-scale insights into the human neuromuscular junction
Source: Cell Rep Methods. 2025 Jun 16;5(6):101082. doi: 10.1016/j.crmeth.2025.101082 (PMC12272245; doi:10.1016/j.crmeth.2025.101082)
Supplement: Document S1. Figures S1–S6 [file mmc1.pdf]

Cell Reports Methods, Volume *5*

Supplemental information

Expansion microscopy reveals nano-scale insights into the human neuromuscular junction

Abdullah Ramadan, Thomas M.D. Sheard, Abrar Alhindi, Philippa A. Rust, Ross A. Jones, Izzy Jayasinghe, and Thomas H. Gillingwater

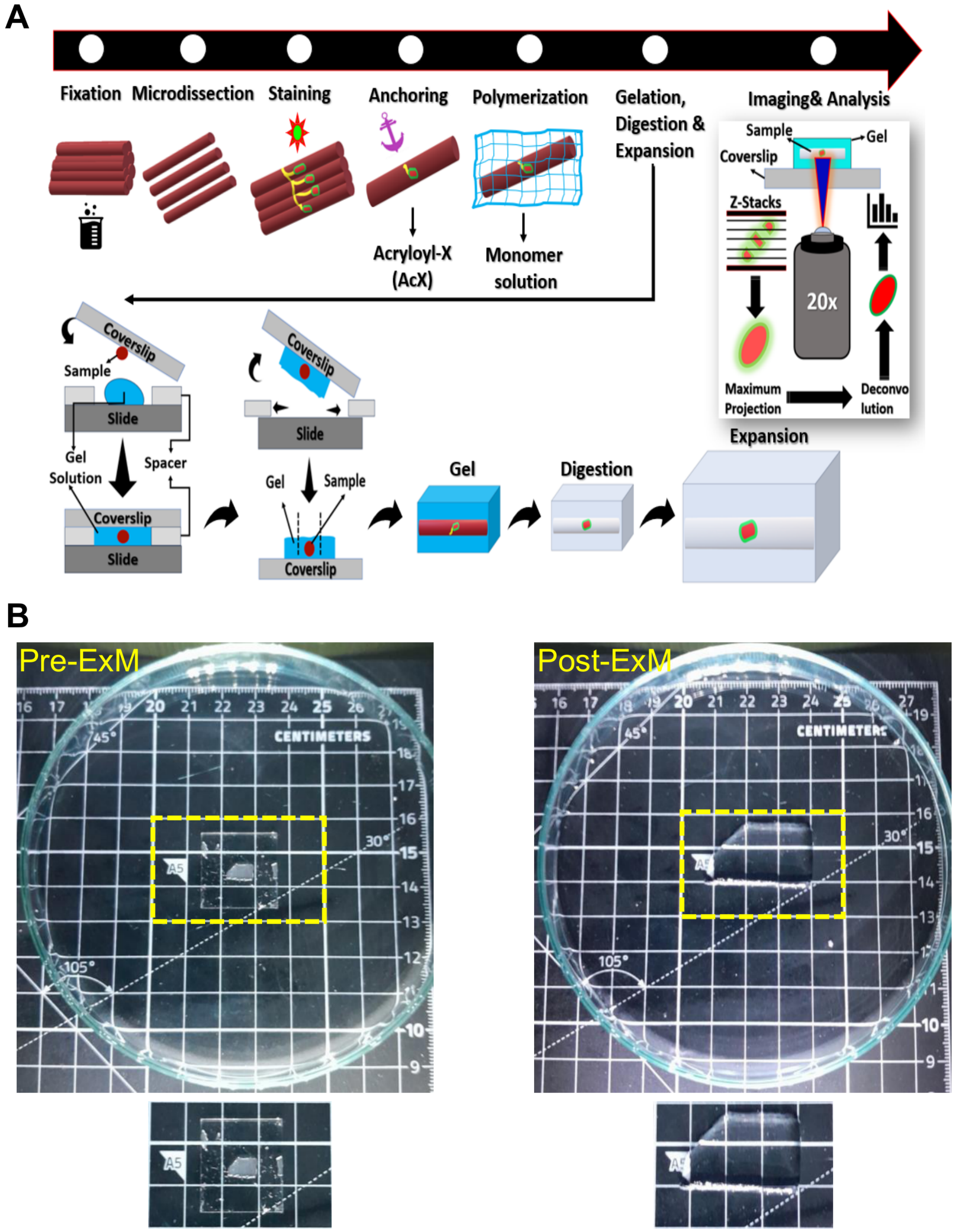

Figure S1: Expansion microscopy workflow and gel expansion in action, related to STAR Methods. (A) Schematic illustration of the workflow of the ExM NMJ protocol, which takes ~5 days to complete. At the beginning and after appropriate fixation of the muscle tissue with 4% PFA and fine dissection, samples are labelled/stained using conventional NMJ staining protocols (see Methods). Subsequently, samples are treated with Acryloyl-X SE (AcX) to anchor fluorescent proteins to the hydrogel before a monomer solution is applied to the sample. Following this, polymerization of the sample and formation of the hydrogel occur in the gel chamber between a coverslip (on top) and a slide (underneath). Digestion using proteinase K with digestive buffer is then applied to clear the specimen from any remaining tissue that would impede the even expansion of fluorescent proteins. Finally, samples can be expanded in ddH20 before imaging using a standard confocal microscope. **(B)** Representative photographs of the expansion process of a hydrogel loaded with muscle/NMJ samples. **(Left)** The gel pre-ExM on a petri dish. **(Right)** The gel expanded ≈4x. Note that the gel appears transparent and is evenly expanded with no signs of distortion. In this example the distances as follow: the X-axis in the pre-expansion status is 7.5 and after expansion is 30 mm. The pre-expansion distance of the Y-axis is 4.5 and 18 mm in post-expansion status.

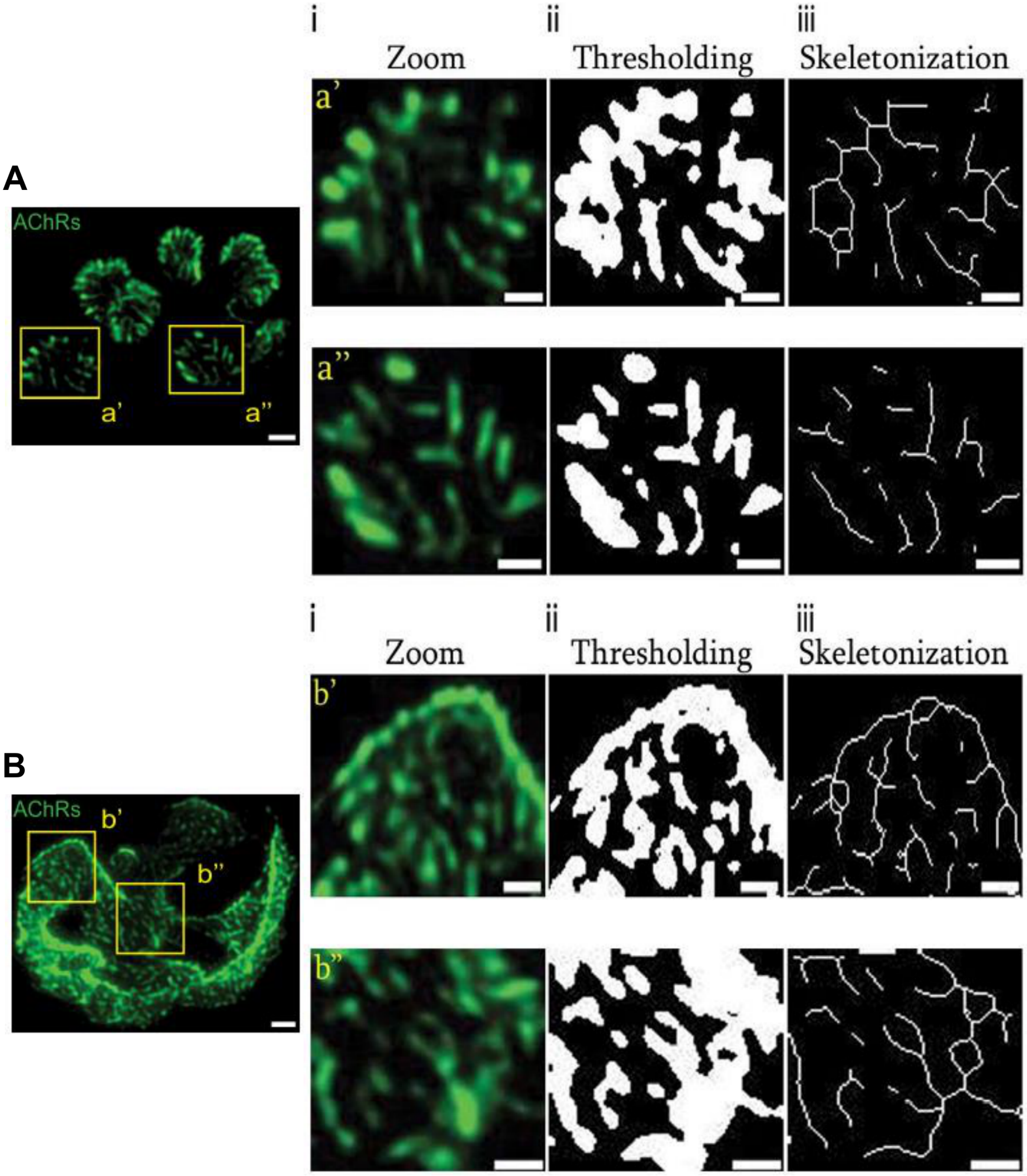

Figure S2: Image processing methodology for quantifying AChR distribution on ExM images, related to Figure 5. Example images of single synaptic boutons from a human NMJ **(A)** and an equivalent area from a mouse NMJ **(B)**. After zooming in **(i)**, measurement of AChR width was performed on thresholded images **(ii)**, whilst length and distance between stripes was measured using on skeletonization-processed images **(iii)**. Scale bars= 4μm corresponding to the pre-expansion dimension, while the zoomed-in images =2 μm pre-expansion dimension.

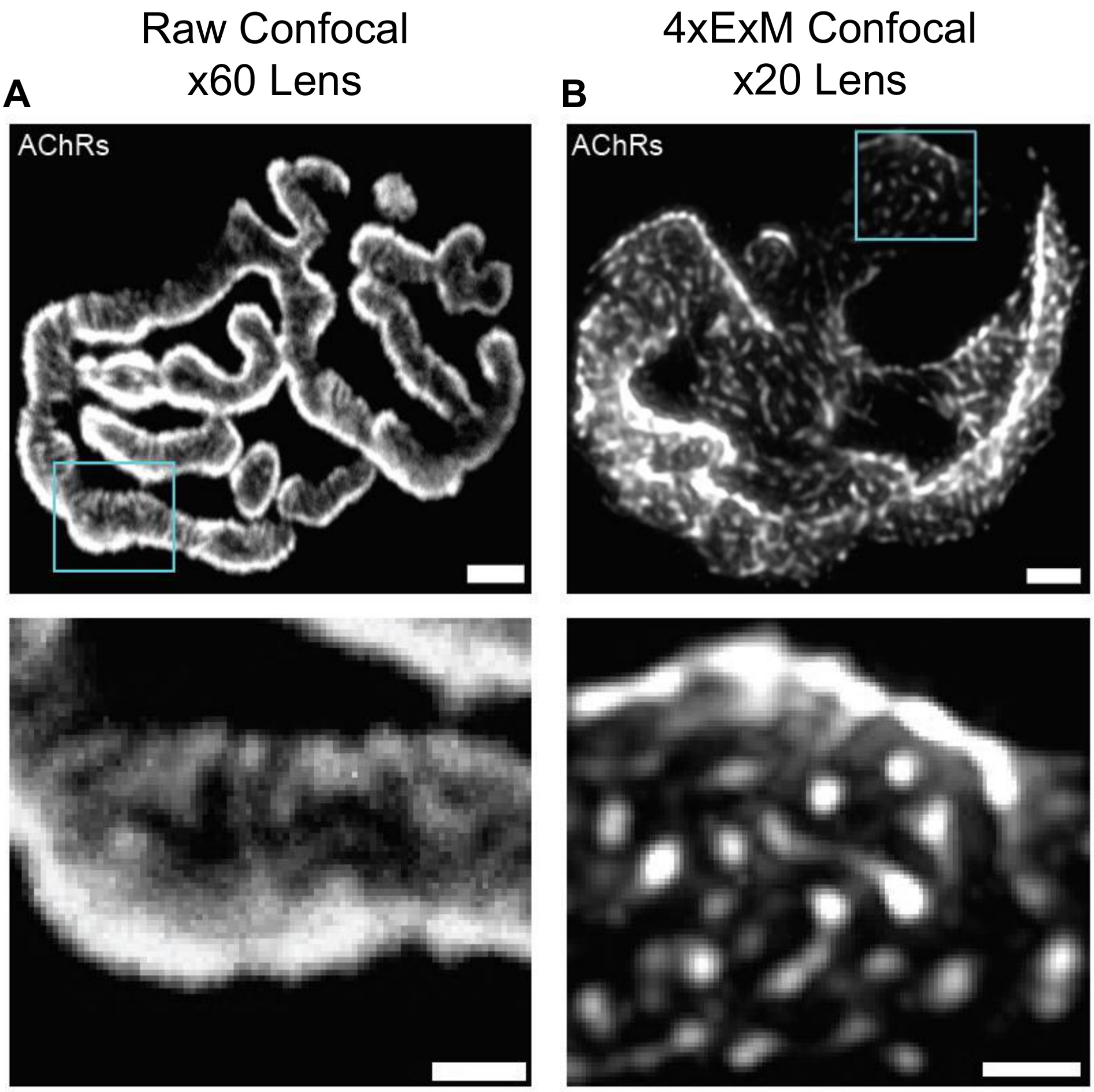

Figure S3: Increased resolution of mouse NMJ structure using ExM compared to standard confocal microscopy, related to Figure 3. Representative examples showing the superiority of 4xExM to standard confocal microscopy with respect to revealing NMJ ultrastructure in mouse tissues. **(A)** representative examples of raw confocal images of AChRs at a single mouse NMJ, acquired using a x60 objective, whereas **(B)** is an image of AChRs at an expanded (4x) NMJ captured using x20 objective. Scale bars =4 µm (in biological units =~1 µm pre-expansion). ExM images revealed significantly more details due to the increased spatial separation of fluorophores in the ExM protocol. When zooming in on a single bouton (bottom images), the expanded NMJs maintained good image quality and resolution, allowing for the identification and tracing of details that were not possible with raw confocal images, which lost resolution and became blurred upon zooming in. Scale bars in zoomed-in images =2 µm (in biological units = 500 nm).

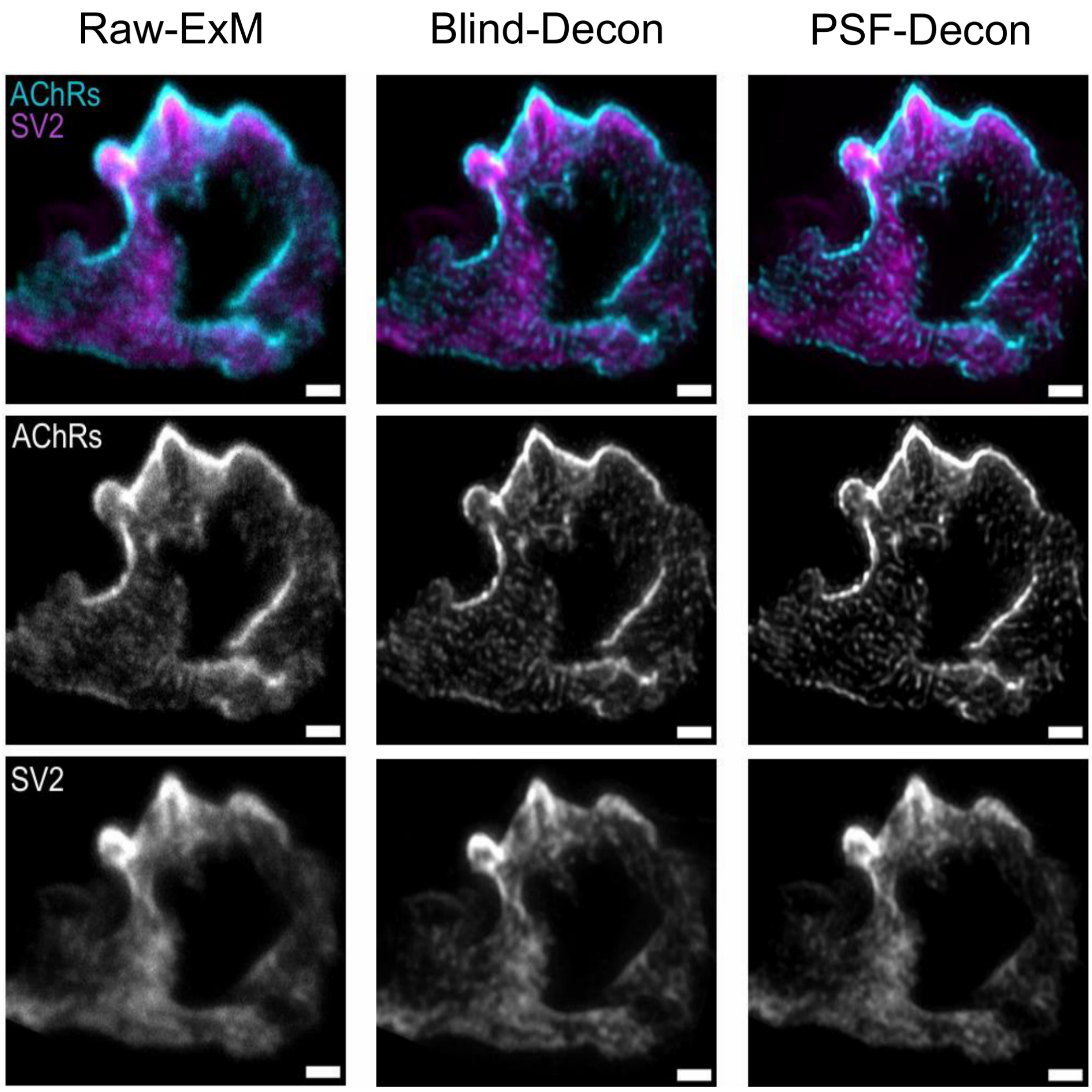

Figure S4: Comparison of different deconvolution techniques for use on ExM images, related to Figure 4. Series of images illustrating the effect of deconvolution on images of expanded (≈ x4) Mouse NMJs. Left panels show an unprocessed raw image, with middle columns showing the same images after applying the Classical Maximum Likelihood Estimation (CMLE) deconvolution algorithm based on the theoretical Point Spread Function (PSF), and the right columns showing the same images processed using an identical deconvolution algorithm but with the precise experimental PSF. NMJs were stained with α-Bungarotoxin for AChRs (in cyan) and SV2 for presynaptic vesicles (magenta). Notably, the deconvolution process led to a substantial enhancement in image contrast, noise reduction, and resolution in visualizing nano-scale structural details. Scale bars = 4 μm, (biological unit =1 μm pre-expansion).

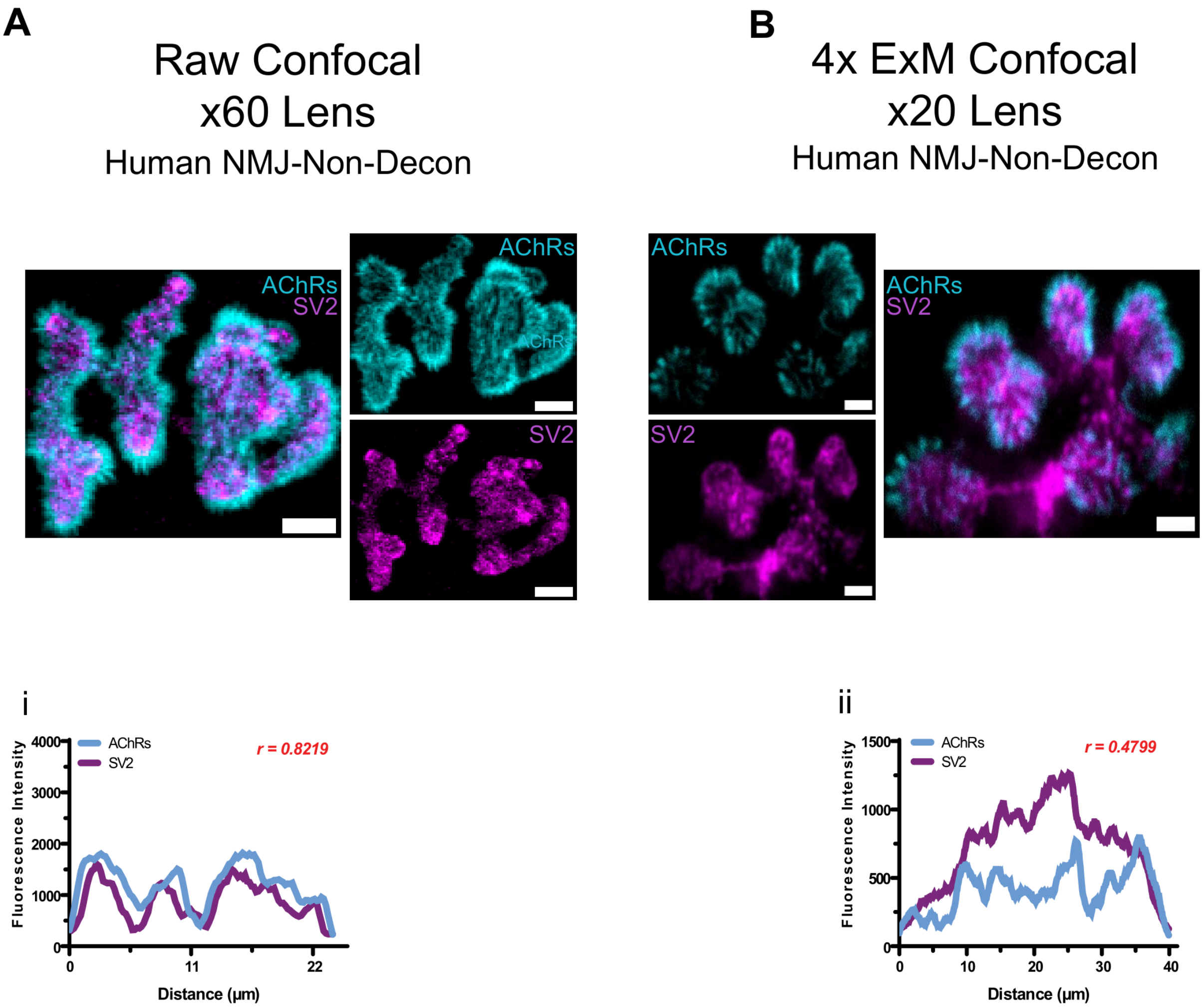

Figure S5: Differential alignment of pre-and post-synaptic structures at the human NMJ revealed by ExM is not detectable using standard confocal microscopy, related to Figure 6 and 7. Comparison of AChR (cyan) and SV2 (magenta) labelling at human NMJs imaged using **(A)** standard confocal microscopy pre-expansion or **(B)** 4x-ExM. Note that the punctate SV2 distribution was not as clear on the standard confocal micrographs. Quantitative analysis of fluorescence intensity profiles revealed no spatial separation in the standard preparations (Pearson correlation of 0.8219) **(i)**, in contrast to the greater spatial dissociation between SV2 and AChRs in the fluorescence intensity profiles from ExM images (Pearson correlation of 0.4799) **(ii)**. Scale bars = 4µm, corresponding to pre-expansion dimensions.

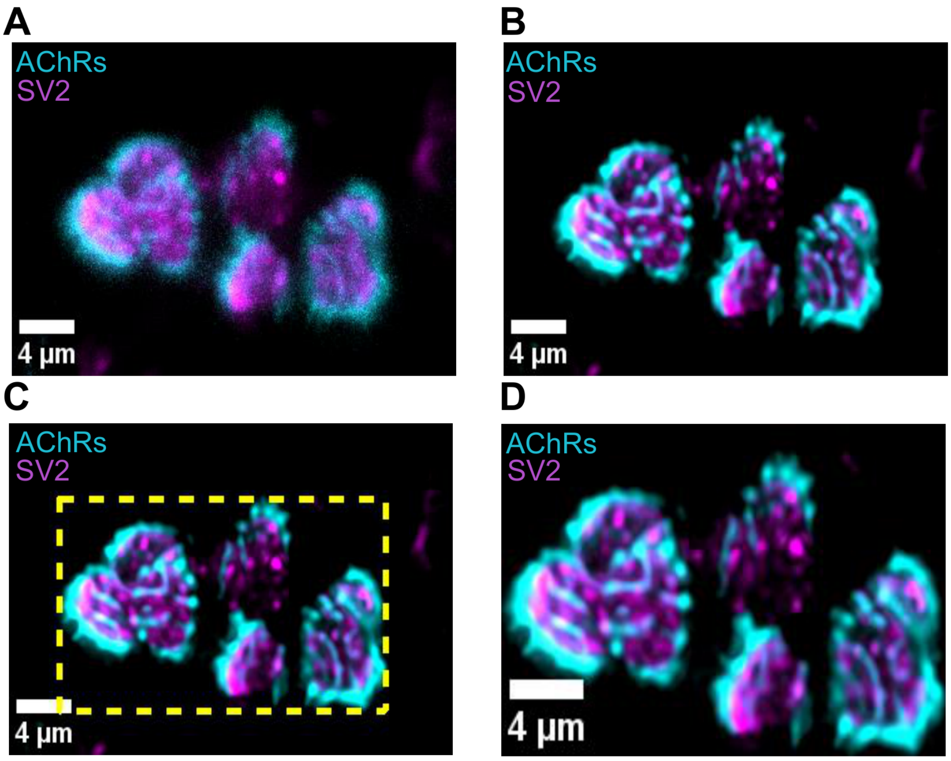

Figure S6: Fluorescence intensity profile selection example, related to Figure 6 and 7. Representative micrographs of ≈x4ExM NMJs revealing AChR distribution (cyan) versus SV2 distribution (magenta) in human NMJ. **(A)** shows the original confocal micrograph. **(B)** the same image after deconvolution. **(C)** The yellow-dotted quadrangle in **(B)** represents the selected region where the quantitative analysis profile was run. **(D)** the final cropped image as appears in the manuscript which reflects the entire NMJ, excluding the non-fluorescent area (black area) where the analysis was run and the Pearson correlation was calculated accordingly. Scale bars = 4µm.
