# Supplementary material for: Expansion microscopy reveals nano-scale insights into the human neuromuscular junction
Source: Cell Rep Methods. 2025 Jun 16;5(6):101082. doi: 10.1016/j.crmeth.2025.101082 (PMC12272245; doi:10.1016/j.crmeth.2025.101082)
Supplement: Document S2. Article plus supplemental information [file mmc3.pdf]

Cell Reports Methods

Article

Expansion microscopy reveals nano-scale insights into the human neuromuscular junction

Graphical abstract

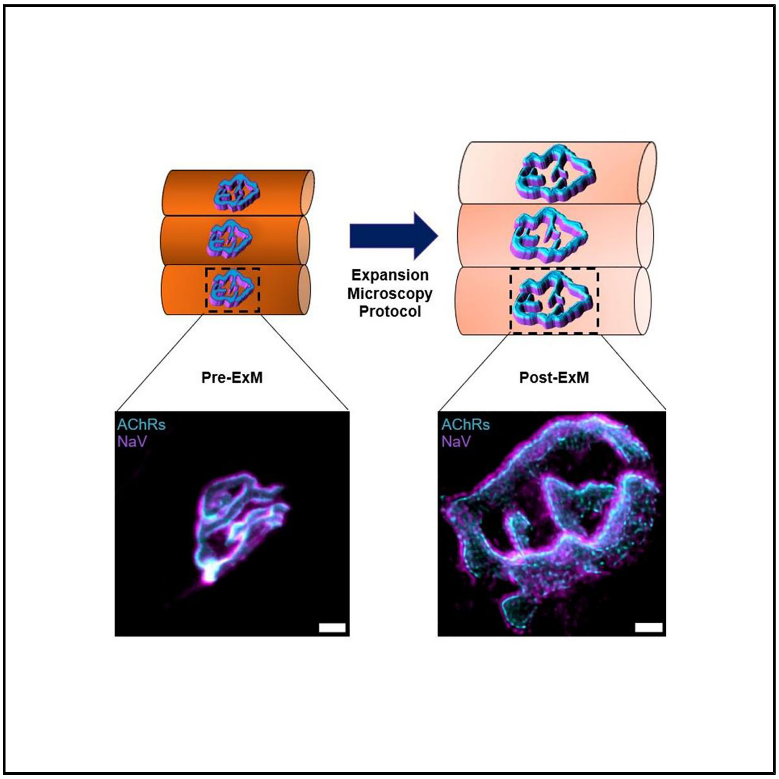

Authors

Abdullah Ramadan, Thomas M.D. Sheard, Abrar Alhindi, Philippa A. Rust, Ross A. Jones, Izzy Jayasinghe, Thomas H. Gillingwater

Correspondence

t.gillingwater@ed.ac.uk

In brief

Ramadan et al. employ expansion microscopy to visualize neuromuscular junctions in human and mouse muscles. Their findings demonstrate expansion microscopy as an accessible and cost-effective approach for high-resolution structural analysis of neuromuscular junctions and reveal species-specific differences in acetylcholine receptor patterning, synaptic vesicle distribution, and NaV1.4 localization.

Highlights

- Expansion microscopy (ExM) can be adapted for use in skeletal muscle
- ExM enables nano-scale imaging of neuromuscular junctions (NMJs)
- ExM can be used for imaging both human and mouse NMJs
- ExM reveals morphological differences between human and mouse NMJs

Ramadan et al., 2025, Cell Reports Methods *5*, 101082
June 16, 2025 © 2025 The Author(s). Published by Elsevier Inc.
https://doi.org/10.1016/j.crmeth.2025.101082

CellPress

Cell Reports Methods

CellPress
OPEN ACCESS

Article

Expansion microscopy reveals nano-scale insights into the human neuromuscular junction

Abdullah Ramadan,[1,2] Thomas M.D. Sheard,[3] Abrar Alhindi,[4] Philippa A. Rust,[1,5] Ross A. Jones,[1,6] Izzy Jayasinghe,[3,7] and Thomas H. Gillingwater[1,6,8,*]
[1]Edinburgh Medical School: Biomedical Sciences, University of Edinburgh, Edinburgh, UK
[2]College of Sciences and Health Professions, King Saud Bin Abdulaziz for Health Sciences, Jeddah, Saudi Arabia
[3]School of Biosciences, Faculty of Science, University of Sheffield, Sheffield S10 2TN, UK
[4]Clinical Anatomy Department, Faculty of Medicine, King Abdulaziz University, Jeddah, Saudi Arabia
[5]Hooper Hand Unit, St John's Hospital, Howden Road West, Livingston, UK
[6]Euan MacDonald Centre for Motor Neuron Disease Research, University of Edinburgh, Edinburgh, UK
[7]EMBL Australia Node in Single Molecule Science, Department of Molecular Medicine, School of Biomedical Sciences, UNSW Sydney, Sydney, NSW, Australia
[8]Lead contact
*Correspondence: t.gillingwater@ed.ac.uk
https://doi.org/10.1016/j.crmeth.2025.101082

MOTIVATION The nano-scale structure of human neuromuscular junctions (NMJs) has been difficult to assess using super-resolution imaging approaches due to practical and technical limitations associated with muscle biopsy specimens, as well as the high costs and specialized equipment required to perform super-resolution microscopy. To address this issue, we have developed and optimized expansion microscopy procedures suitable for use with human and mouse muscle tissues. We subsequently developed specialized morphological quantification approaches to compare nano-scale features of NMJs between species.

SUMMARY

The neuromuscular junction (NMJ) is a specialized synapse that relays signals from the lower motor neuron to the skeletal muscle. Here, we detail the development and application of expansion microscopy (ExM) as a highly accessible, relatively cheap, powerful, and reproducible tool with which to obtain high-resolution insights into the subcellular structure and function of NMJs from whole-mount preparations, previously only achievable using super-resolution microscopy. ExM is equally applicable to both mouse and human tissue samples, facilitating high-resolution comparative analyses. Qualitative and quantitative analysis of ExM images reveals significant differences in the distribution of acetylcholine receptors, synaptic vesicles, and voltage-gated Na^+ 1.4 (NaV1.4) channels between human and mouse NMJs that are not readily observable using conventional confocal microscopy. We conclude that ExM offers a cost-effective and adaptable approach to facilitate nano-scale imaging of the NMJ.

INTRODUCTION

The neuromuscular junction (NMJ) is a large chemical synapse that relays signals from a presynaptic lower motor neuron to a postsynaptic skeletal muscle fiber. Here, acetylcholine (ACh) released from motor nerve terminals signals to specialized sites on the sarcolemma, referred to as motor endplates, enriched with ACh receptors (AChRs).[1] The sarcolemma of the motor endplate is invaginated to create a series of junctional folds; AChRs are distributed on the crest of the folds, while voltage-gated Na^+ 1.4 (NaV1.4) channels are located in the troughs between the bases of the folds.[2] The NMJ has garnered a great deal of attention in neuroscience research for over 100 years, serving as an experimentally accessible model to study fundamental principles of synaptic biology.[3] Recent insights revealing the NMJ to be a primary and early site of pathology in many neurodegenerative conditions[4–6] have highlighted the importance of understanding NMJ form and function both in health and during disease. Although NMJs in most vertebrate species share essential characteristics, notable differences do exist. For example, the human NMJ presents with a small, nummular morphology, while the mouse NMJ displays a large, pretzel-shaped arrangement.[7] In addition, the human NMJ does not demonstrate any of the morphological changes associated with aging described in rodents, where the NMJ gradually fragments and becomes denervated.[7]

© 2025 The Author(s). Published by Elsevier Inc.
This is an open access article under the CC BY license (http://creativecommons.org/licenses/by/4.0/).

Fluorescence microscopy has proven to be an important tool for the study of NMJs and biological systems in general, providing insights into both structure and function.[8] Over the past couple of decades, significant advancements in camera technology, light sources, and fluorophores have led to the development of super-resolution (SR) microscopy techniques,[9] such as stimulated emission depletion (STED) microscopy, stochastic optical reconstruction microscopy (STORM), and structured illumination microscopy (SIM), which enable the visualization of nano-scale structures by overcoming the diffraction limit of light.[7,10–12] With respect to NMJ studies in mice, SIM and 3D-STORM have been used to demonstrate the aggregation of AChRs at the edges of junctional fold crests.[12] Similarly, the spatial relationship and distribution pattern of active zones have been studied using STED microscopy.[11] In contrast, there has only been limited application of SR imaging for studying human NMJs.[7]

Although SR imaging methods have an important role to play in the neuroscience field, limitations include the need for specialized equipment, restricted fluorophore options, increased risk of phototoxicity, slow imaging speeds, high costs, and operational complexity.[13,14] Most localization-based SR imaging methods are particularly limited by the poorer axial resolution (>>100 nm) as well as the background fluorescence resulting from the three-dimensionally complex tissue samples,[15] requiring the tissue to be thinly sectioned. One recently developed alternative to these approaches that offers similar nano-scale insights, but without the associated limitations, is expansion microscopy (ExM). ExM uses novel sample preparation protocols to label molecules in fixed cells/tissues that can be anchored in a hydrogel; the original biological specimen is chemically digested and then the gel is physically enlarged using osmotic swelling. This permits individual molecules and organelles to be resolved from one another using a conventional microscope setup (e.g., confocal microscopy) even if they were below the diffraction limit of light beforehand.[15,16] ExM therefore permits a user to acquire SR and high-resolution images but using standard fluorophores and conventional microscopes,[13] making it both easier to implement and more cost effective.[15]

Here, we set out to establish whether ExM could be utilized and adapted for the study of NMJs, with a particular aim to use it for SR studies of human NMJs, moving beyond the level of detail that standard confocal microscopy can provide. We report on the successful modification of a 4×ExM protocol, revealing nano-scale architectural details of human and mouse NMJs from whole-mount samples that did not require prior tissue sectioning. We describe a detailed workflow for applying the ExM technique to study NMJs using a conventional confocal microscope with 20× objective lens magnification and demonstrate its potential to reveal species-specific nano-scale features of NMJs in humans.

RESULTS

Establishing an ExM protocol for nano-scale imaging of the NMJ in whole-mount preparations

In order to establish an NMJ-specific ExM protocol suitable for surgical biopsy samples of skeletal muscle from human patients, we started by adapting an existing 4×ExM protocol initially developed to study cardiac ryanodine receptor clusters in cardiomyocytes *in vitro*[17] (Figure S1).

The first critical modifications were applied to convert the technique to be feasible for use with whole-mount muscle biopsy samples, which are required for successful localization and visualization of NMJs. We avoided sectioning of whole-mount muscle biopsy samples due to their relative size and the need to maintain tissue architecture. In ExM experiments, gelation with a sample typically occurs on a microscope slide in a confined chamber whose size is defined by glass coverslip spacers.[18] For free-floating samples not attached to a surface like a coverslip, the introduction of gel solution can disturb the orientation of the sample, thus hampering the identification of the NMJ bands and causing the muscle bundle to lose its gross architecture (e.g., folding over itself) or to float within the solution. Moreover, during the gelation process, this approach led to uneven gel formation, causing cracking and visible distortion during expansion. To address this issue, we transferred muscle biopsy samples onto a coverslip coated with poly-L-lysine. This enabled the orientation of the muscle biopsy sample to be controlled within the gel.

The second major protocol modification that was required was an optimized digestion step.[16] In the present study, we found that the application of digestive buffer for 18–20 h at 37°C was effective in clearing the biological tissue within the gel while maintaining a sufficient fluorescent signal for subsequent imaging (see STAR Methods). Insufficient digestion in a shorter time or lower temperature resulted in visible gel distortions, but at the same time, the digestion step should be restricted in order to prevent the loss of fluorescence. The remainder of the protocol was performed as described previously.[17]

In order to assess the validity and applicability of the newly developed ExM protocol for visualizing NMJs in whole-mount preparations, we initially examined fluorescently labeled NMJs in surgical biopsy samples from the dorsal interosseus muscle of human patients and comparable forelimb interosseous muscles from young adult mice (see STAR Methods). To assess both pre- and postsynaptic elements of the NMJ, we used a panel of immunohistochemical and toxin-based labels: AChRs were labeled using Alexa 488-conjugated α-bungarotoxin, with synaptic vesicles or postsynaptic sodium channels labeled with antibodies against synaptic vesicle protein 2 (SV2) or NaV1.4, respectively (see STAR Methods). Tissue samples were processed for 4×ExM using the protocol shown in Figure S1A and visualized using a standard Nikon A1R FLIM confocal laser scanning microscope equipped with 20×/0.75 NA air objective. z stack images were obtained with a 0.5 μm interval at 9× zoom.

The expansion factor and distortion can be assessed through two methods: macroscopic validation and microscopic validation.[19] Macroscopic validation involves measuring the gel before and after expansion while accounting for any visible deformity. Microscopic validation entails measuring the same well-defined biological structure within the gel before and after expansion. To validate macroscopic expansion, we relied on the overall gel dimensions and shape (Figure S1B). For micron- and sub-micron-scale verification of expansion isotropy, we compared the end-plate area averages between expanded and non-expanded NMJs (as Figure 2 shows). Also, the current literature indicates that 4×ExM does not cause a vast distortion or uncorrected

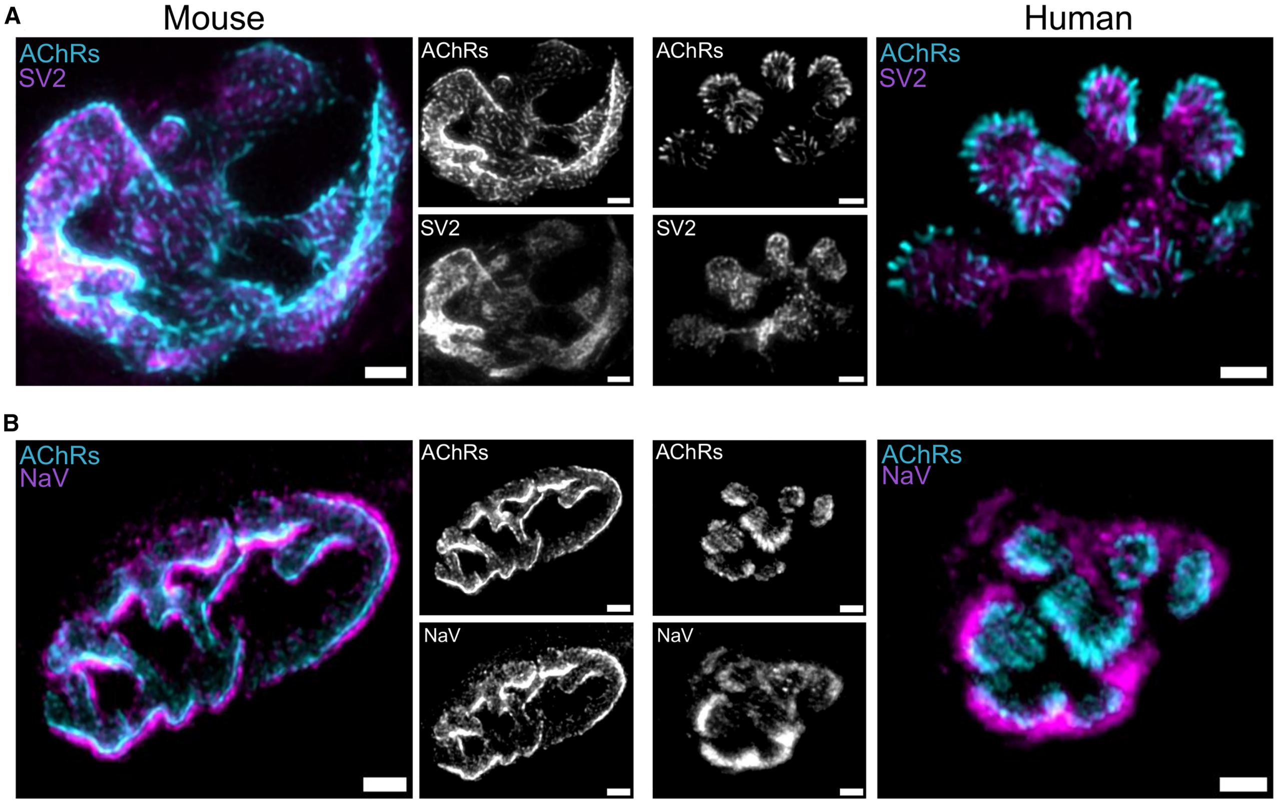

Figure 1. Human and mouse NMJs imaged using ExM

(A) Representative confocal micrographs showing examples of expanded NMJs (≈4×) from mouse (left) and human (right) muscle biopsies stained with α-bungarotoxin to reveal AChRs (cyan) and immunohistochemical labeling of SV2 to reveal presynaptic vesicles (magenta). Note the level of detail that can be observed with regard to the precise distribution and morphological arrangement of each label: AChRs exhibited distinctive patterns between human and mouse NMJs and SV2-labeled synaptic vesicles exhibited a more punctate distribution at the human NMJ.

(B) Representative confocal micrographs showing examples of expanded NMJs (≈4×) from mouse (left) and human (right) muscle biopsies stained with α-bungarotoxin to reveal AChRs (cyan) and immunohistochemical labeling of NaV1.4 to reveal Na channels (magenta). Again, note the level of detail that can be observed with regard to the precise distribution and morphological arrangement of each label: in human NMJs, the boundaries of Nav1.4 rims extended further beyond the AChRs compared to those in mice.

Images in (A) and (B) were acquired with a 20× objective and treated with a deconvolution algorithm. The scale bars (5 μm) correspond to pre-expansion dimensions.

microscopic expansion factor compared with the macroscopic validation.[19–21] Microscopic validation was impracticable in our study due to two primary challenges. Firstly, difficulties in imaging and tracking the same NMJ before and after gelation were caused by altering muscle fiber orientation during manipulation and transfer. Secondly, the 3D complexity and thickness of the sample did not allow for maintaining the same focal plane before and after the expansion. For example, the diameter of a single muscle fiber (e.g., ∼70 μm in human or ∼50 μm in mouse) would be extended across an imaging depth of 200–300 μm following gel expansion, while the focal volume in one confocal plane would be proportionally shallower.

The overall gross appearance of expanded NMJs was entirely consistent with the myriad examples of non-expanded NMJ images in the current literature obtained by conventional confocal microscopy. Thus, NMJs still possessed the usual coin- or pretzel-shaped morphology from humans or mice, respectively, and with a motor nerve terminal closely aligned to the neighboring motor endplate on the muscle fiber (Figure 1). There was no evidence of obvious distortion of the pre- and/or postsynaptic aspects of the NMJ during the expansion process, consistent with what was previously reported: that conventional ExM protocols do not induce any significant distortion that could otherwise impact quantitative analysis of expanded structures.[19,20] Likewise, we observed no evidence of distortion either macroscopically, where gels were noted to have expanded with preserved macroscopic shape (Figure S1B), and/or in the consistency of NMJ morphology in their pre-ExM and post-ExM states (Figure 2A). Quantitative assessment of NMJ size—using the endplate area as a proxy measurement—in pre- and post-expanded samples confirmed a significant, ∼4× enlargement in both human and mouse samples (Figure 2B). Thus, the size corrected for the expansion factor (corrected size) of the mouse NMJ was increased from ∼250 to ∼1,000 μm^2 post-ExM. Similarly, the actual size of the human NMJ was increased from ∼130 to ∼600 μm^2 post-ExM.

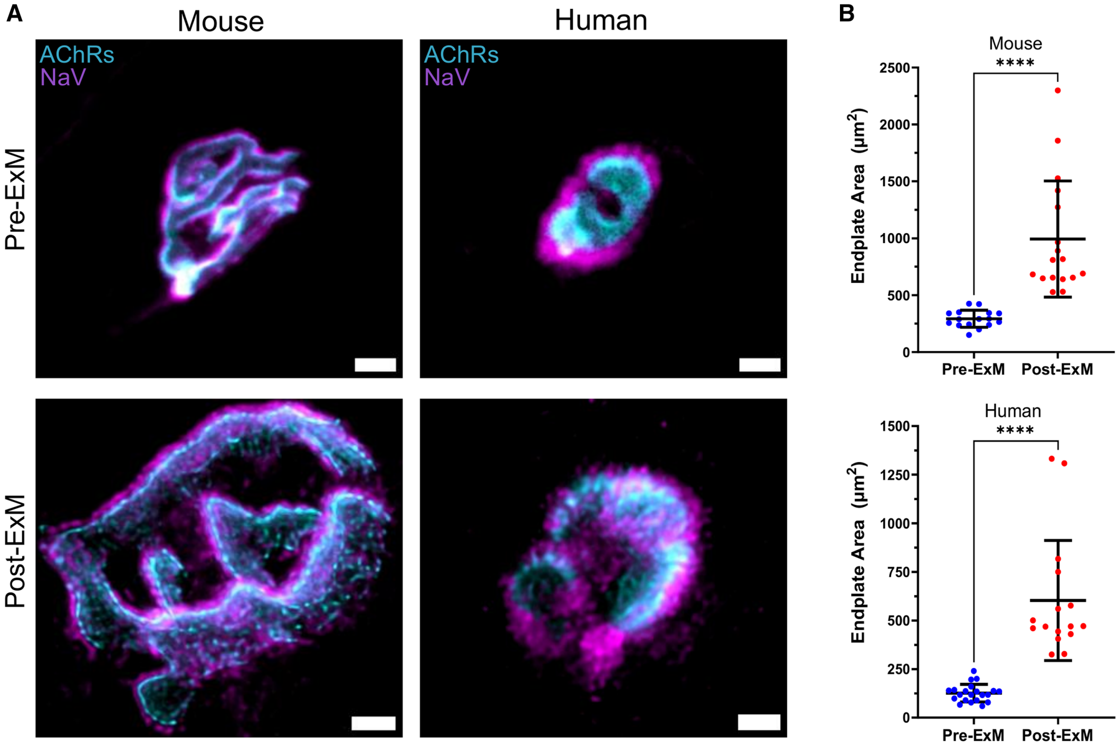

Figure 2. Pre- and post-expansion analysis of NMJs

Comparison of representative confocal micrographs of NMJs, pre- and post-expansion.

(A) Examples of mouse (left) and human (right) NMJs pre- and post-expansion (top and bottom, respectively). Note: these are examples of different NMJs, as it was not possible to image the same NMJs before and after the expansion process. NMJ samples were labeled and imaged identically: α-bungarotoxin for AChRs (cyan) and NaV1.4 for Na channels (magenta) as two distinct dual-pseudo colors; images were acquired with a 20× objective. Scale bars (4 μm) correspond to pre-expansion dimensions.

(B) Quantitative analysis of NMJ endplate area as a core morphological variable pre- and post-expansion in both mouse (top) and human (bottom) NMJs revealed an expansion factor of ~4× with ExM. Bar charts represent mean ± SD; each data point represents an individual NMJ (mouse pre-ExM n = 16 and post-ExM n = 17, while human pre-ExM n = 21 and post-ExM n = 17). Unpaired t test (two-tailed), ****$p < 0.0001$.

High-magnification objectives (>20×) offer enhanced resolution and superior visualization; however, their limited working distances present a significant limitation.[18] This issue renders them unsuitable for ExM in our samples, which are derived from thick muscular tissues of both human and mouse origins. Notably, the NMJ has a thickness ranging from 20 to 30 μm in its unexpanded state, situated on muscle fibers with an approximate diameter of 80 μm. These dimensions increase substantially following the application of the ExM protocol, as the z stack depth expands during imaging. Based on our observations, the use of a 40× oil-immersion objective lens (or higher magnifications) is impractical for imaging thick expanded muscular tissues, particularly in z stack (3D) imaging. Consequently, we conclude that a 20× air objective lens is the most suitable option for such experimental work, given the nature of these samples and the imaging requirements.

Application and validation of ExM for nano-scale imaging of the NMJ

Having developed a robust ExM protocol that can be successfully used to visualize both human and mouse NMJs, we next observed the complexities of the structural features revealed by ExM, as compared with conventional confocal microscopy. It is well known that the distribution of AChRs within the human NMJ is not uniform,[12] although the precise spatial organization remains indistinct when employing conventional confocal imaging (Figure 3A), with similar issues for mouse NMJs (Figure S3A). In contrast, the modified 4×ExM technique produced images with far greater resolution, revealing a more refined spatial distribution of AChRs, even when employing a lower-magnification objective lens, for both human (Figure 3B) and mouse (Figure S3B) NMJs. This enhancement clearly demonstrated the underlying principles of ExM, whereby the increased spatial separation of fluorophores within the fixed, stained sample facilitated the enhanced resolution of nano-scale structures.

A comparison of AChR distribution and localization obtained using ExM with AChR distribution and localization obtained using other SR imaging techniques[12] confirmed that ExM was capable of delivering similar levels of high-resolution detail. AChR labeling revealed by using ExM exhibited a distinctive "striped" pattern in both human and mouse NMJs. In human NMJs, these AChR stripes appeared larger in size but fewer in number in comparison with their mouse counterparts (Figures 1A and 1B). These findings are consistent with a

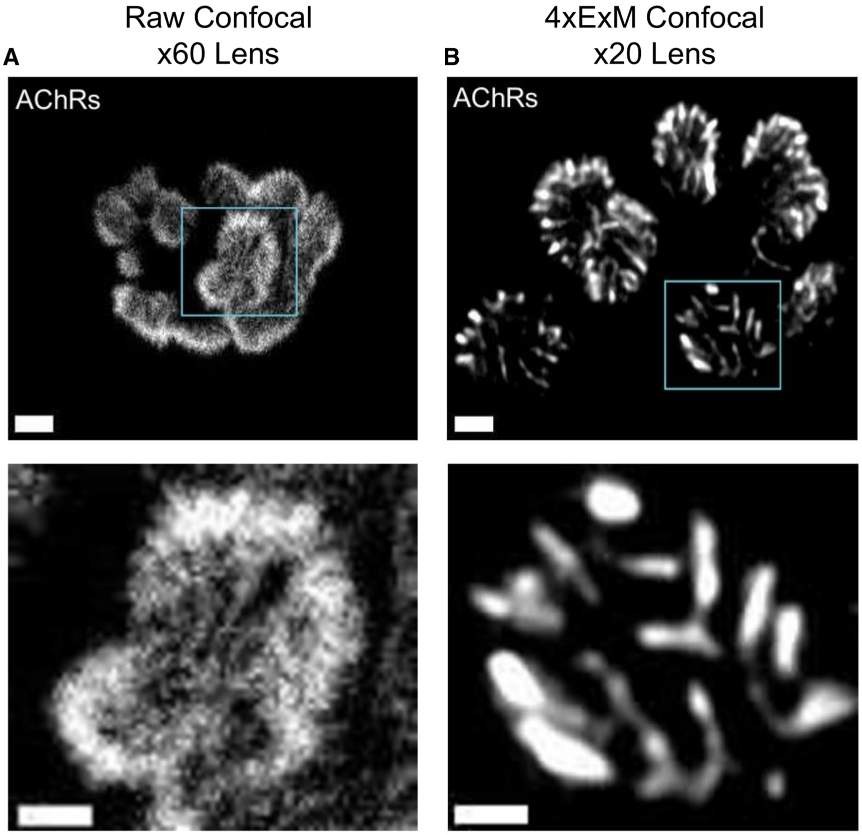

Figure 3. Increased resolution of NMJ structure using ExM compared to standard confocal microscopy

Representative examples showing the superiority of 4×ExM to standard confocal microscopy with respect to revealing NMJ ultrastructure. (A) shows representative examples of raw confocal images of AChRs at a single human NMJ, acquired using a 60× objective, whereas (B) is an image of AChRs at an expanded (4×) NMJ captured using a 20× objective. Scale bars: 4 μm (in biological units = ∼1 μm pre-expansion). ExM images revealed significantly more details due to the increased spatial separation of fluorophores in the ExM protocol. When zooming in on a single bouton (bottom images), the expanded NMJs maintained good image quality and resolution, allowing for the identification and tracing of details that were not possible with raw confocal images, which lost resolution and became blurred upon zooming in. Scale bars in zoomed-in images: 2 μm (in biological units: 500 nm).

previous SR study that used SIM and 3D-STORM to reveal a similar arrangement of AChRs at the mouse NMJ.[12] Similar SR-equivalent levels of detail were also noted when comparing presynaptic structures, as evidenced by the distribution of SV2 labeling revealed using ExM. At the human NMJ, synaptic vesicle labeling visualized using 3D-STORM microscopy exhibits a ''hotspot''-type arrangement, while at the mouse NMJ, SVPs are much more evenly distributed[7] (Figure 1A).

Further differences were noted when comparing endplate NaV1.4 labeling. Here, ExM NMJ confocal imaging demonstrates that the endplate NaV1.4 channels and AChRs overlap in both human and mouse NMJs, as expected based on previous SR studies of the mouse NMJ.[12] Notably, however, in human NMJs, the boundary of the NaV1.4 labeling extended ∼2× further beyond the footprint of the AChR labeling than it did in mice (Figure 1B), indicating more extensive invagination into the muscle sarcolemma. Taken together, these findings confirm that ExM can generate nano-scale insights comparable with SR approaches and also demonstrate the power of ExM to expand the repertoire of molecules suitable for high-resolution analysis at the NMJ.

Quantitative analyses of AChRs in ExM NMJ images

Following these initial qualitative observations of AChR, SV2, and NaV 1.4 distribution, we next wanted to establish whether ExM can be used to undertake a more detailed, quantitative analysis of both human and mouse NMJs. To facilitate this, we first compared a series of deconvolution transformations in order to improve the signal-to-noise (S-to-N) ratio within the 4×ExM NMJ images. We applied the classical maximum likelihood estimation (CMLE) deconvolution algorithm in Huygens software based on the experimental point spread function (PSF) (for more information, see Scientific Volume Imaging, https://svi.nl/Deconvolution-algorithms). In doing so, we were careful to minimize artificial effects; for example, the experimental PSF from the 20× objective lens was calculated in a condition that mimicked the gel status during imaging, where the fluorescence microspheres are floating in water. In particular, we limited the number of iterations to between 50 and 100 on the basis that we achieved the highest consistency in object retention, S-to-N, and contrast improvement in this range of iterations. As a result of the deconvolution process, there was a substantial improvement in image contrast and noise reduction, with the increased clarity facilitating the visualization of intricate structural details within human (Figure 4) and mouse (Figure S4) NMJs. This was considered appropriate for subsequent quantitative analyses.

We developed a quantification protocol for use on the deconvoluted ExM NMJ images (see STAR Methods). For the analysis of AChR distribution, we measured both the width and length of individual AChR stripes, along with the distance between adjacent AChR stripes. To ensure consistency, all analyses were performed on binarized thresholded images,[7] which were also skeletonized as part of the quantification protocol. At all times, we ensured that there was accurate visual alignment between the thresholded images and the original counterparts to prevent misestimation (Figure S2).

Overall, data were obtained from a total of 33 NMJs (human $N = 16$, mouse $N = 17$). For each NMJ, over 500 individual AChR stripes were measured (human $n \geq 500$, mouse $n \geq 700$).

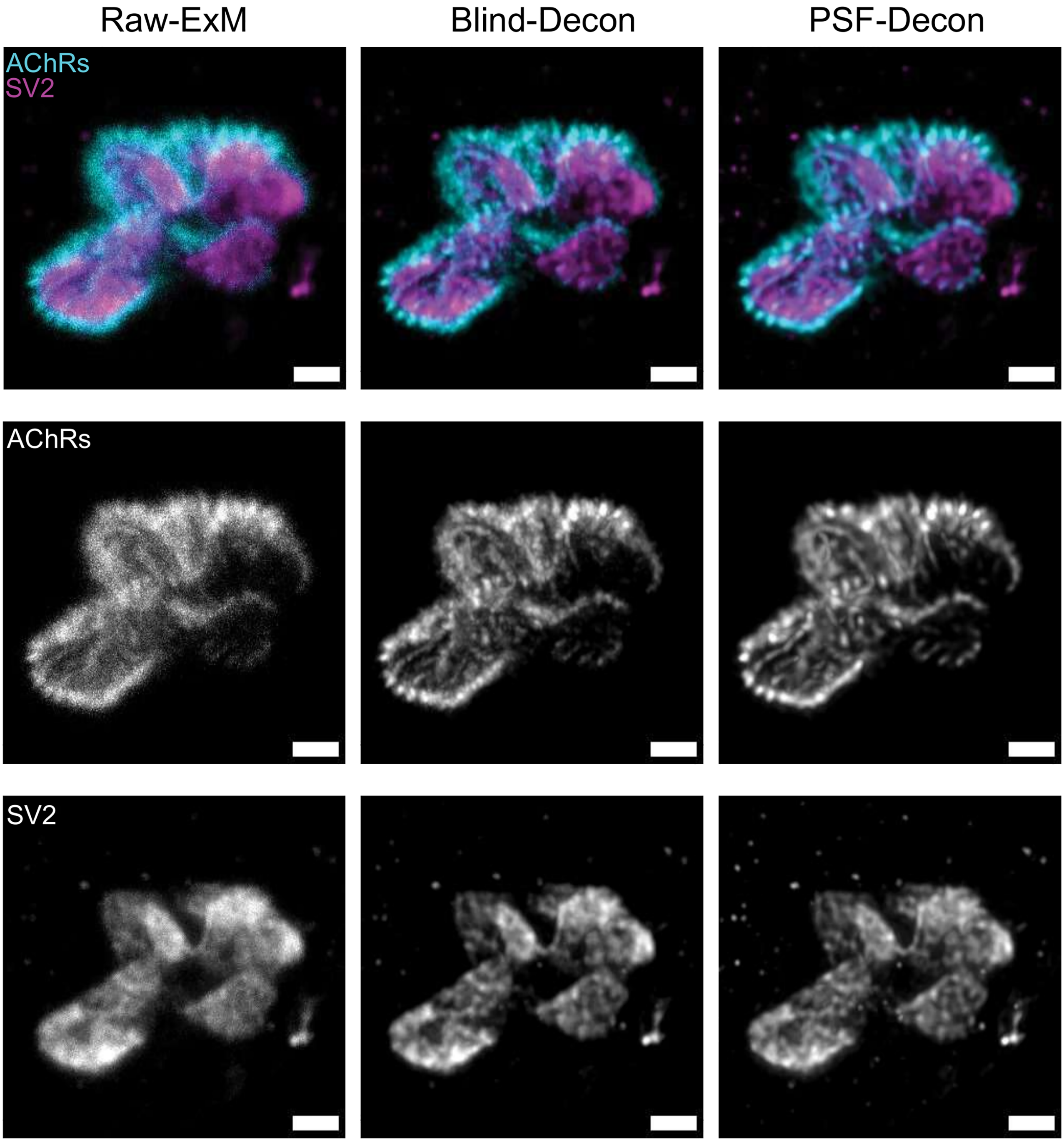

Figure 4. Comparison of different deconvolution techniques for use on ExM images

Series of images illustrating the effect of deconvolution on images of expanded (≈4×) human NMJs. The left images show unprocessed raw images, with middle columns showing the same images after applying the classical maximum likelihood estimation (CMLE) deconvolution algorithm based on the theoretical point spread function (PSF) and the right columns showing the same images processed using an identical deconvolution algorithm but with the precise experimental PSF. NMJs were stained with α-bungarotoxin for AChRs (in cyan) and SV2 for presynaptic vesicles (magenta). Notably, the deconvolution process led to a substantial enhancement in image contrast, noise reduction, and resolution in visualizing nano-scale structural details. Scale bars: 4 μm, (biological unit = 1 μm pre-expansion).

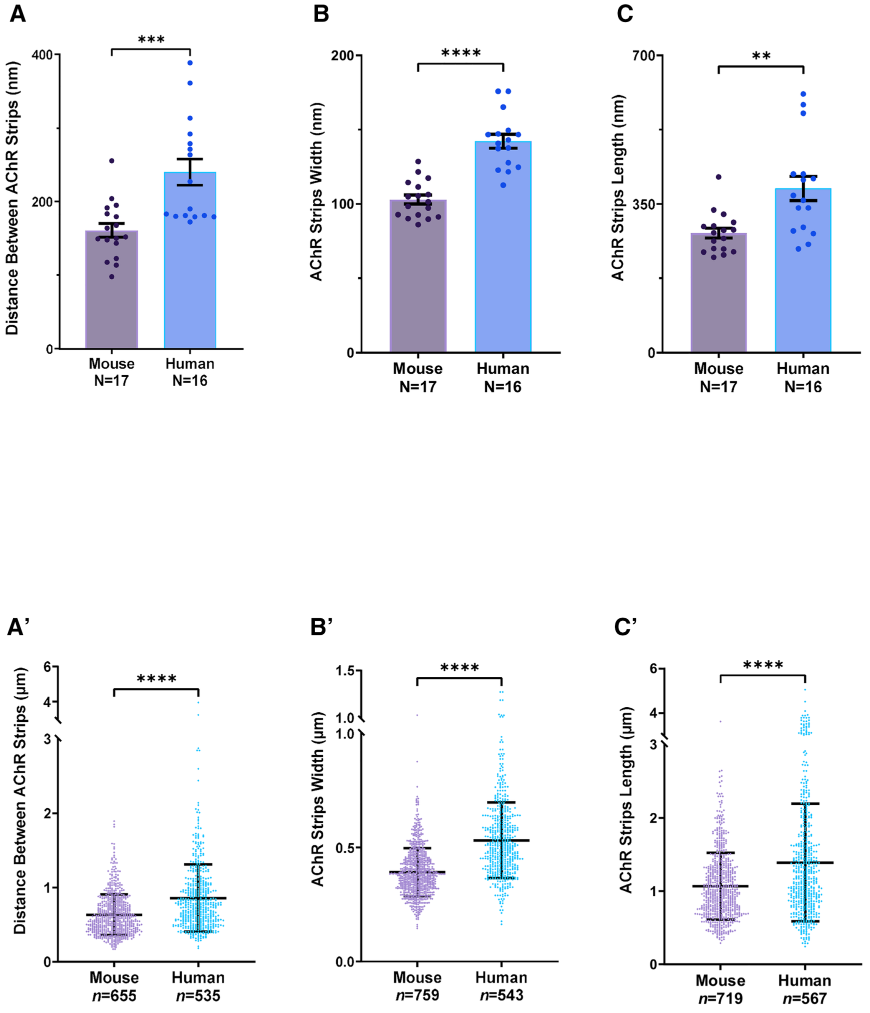

Figure 5. ExM reveals differences in AChR distribution between human and mouse NMJs

(A–C) Quantitative analysis of the morphological characteristics of AChR stripes at human and mouse NMJs imaged using ExM data based on biological data obtained from post-expansion dimensions normalized to the expansion factor. Statistical analysis (unpaired t test) revealed a significant increase in the distance between AChR strips (A), AChR strip width (B), and AChR strip length (C) at the human NMJ compared to mice. Bar charts are presented as mean ± SEM; each data point represents an average of total AChR stripes from a single NMJ (≈35 individual AChR stripes per NMJ in human and ≈35 individual AChR stripes per NMJ in mouse). Total number of human NMJs = 16 (individual AChR stripe $n \approx 500$). Mouse NMJ number = 17 (individual AChR stripe $n \approx 700$). $^{**}p < 0.01$, $^{***}p < 0.001$, and $^{****}p < 0.0001$.

(A′–C′) Absolute dimension data obtained from ExM images, prior to normalization to the expansion factor. Total number of human NMJs = 16 (individual AChR stripes $n \approx 500$). Total number of mouse NMJs = 17 (individual AChR stripes $n \approx 700$). Distance between AChR strips (A′), AChR strip width (B′), and AChR strip length (C′). Bar charts show mean ± SD; each data point represents an individual AChR stripe. Unpaired t test, $^{**}p < 0.01$, $^{***}p < 0.001$, and $^{****}p < 0.0001$.

The quantitative data confirmed initial qualitative appearances, with human NMJs demonstrating AChR stripes that were wider, longer, and more separated from one another than in mouse NMJs. For the purposes of quantification, the absolute dimensions of each variable (in microns) were measured on post-ExM images (Figure 5, bottom); thereafter, these measured values were adjusted by the expansion factor of the gel (×3.8) to derive the true biological dimensions (in nanometers) (Figure 5, top). The corrected values revealed statistically significant differences between human and mouse NMJs across all 3 variables, with human NMJs having mean AChR stripe dimensions of approximately 142 × 387 nm (width × length) with 240 nm spacing, compared with mouse dimensions of 103 × 281 nm with 161 nm spacing (Figure 5). Thus, ExM can be used to obtain detailed quantitative analyses of nano-scale features of human and mouse NMJs.

ExM reveals human-specific morphological features of the NMJ

Next, based on our initial qualitative observations, we wanted to use ExM to explore further the spatial relationship between AChRs at the motor endplate and synaptic vesicles in motor nerve terminals, as well as NaV1.4 channels, at human and mouse NMJs. Physiologically, synaptic transmission commences when action potentials reach nerve terminals, causing a calcium influx and the subsequent fusion of synaptic vesicles with the presynaptic membrane to release ACh[22] at specialized sites: the presynaptic active zone.[23] Previous studies indicated that active zones are directly positioned opposing the openings of postsynaptic infoldings,[12] where AChRs and NaV1.4 channels are located. Certainly, the distinct spatial arrangements of the tri-NMJ components (SV2, AChRs, and NaV1.4) observed between mouse and human NMJs highlight species-specific differences in neurotransmission machinery, as previously suggested.[2,7,24]

Comparative ExM imaging of mouse (Figure 6A) and human (Figure 6B) NMJs revealed species-specific differences in the spatial arrangement and intensity profiles of AChR and SV2 labeling. In mouse NMJs, the signal distribution of the two markers exhibited significant overlap, clearly visible in the ExM images and supported by closely correlated fluorescence intensity profiles (r = 0.81) (Figure 6Ai), indicating a homogeneous distribution of SV2 protein (and hence synaptic vesicles) over the entire region of AChRs at the endplate. In contrast, discernible peaks of SV2 labeling were evident at human NMJs, accompanied by a conspicuous dissociation between SV2 and AChR fluorescence intensity profiles (r = 0.51)

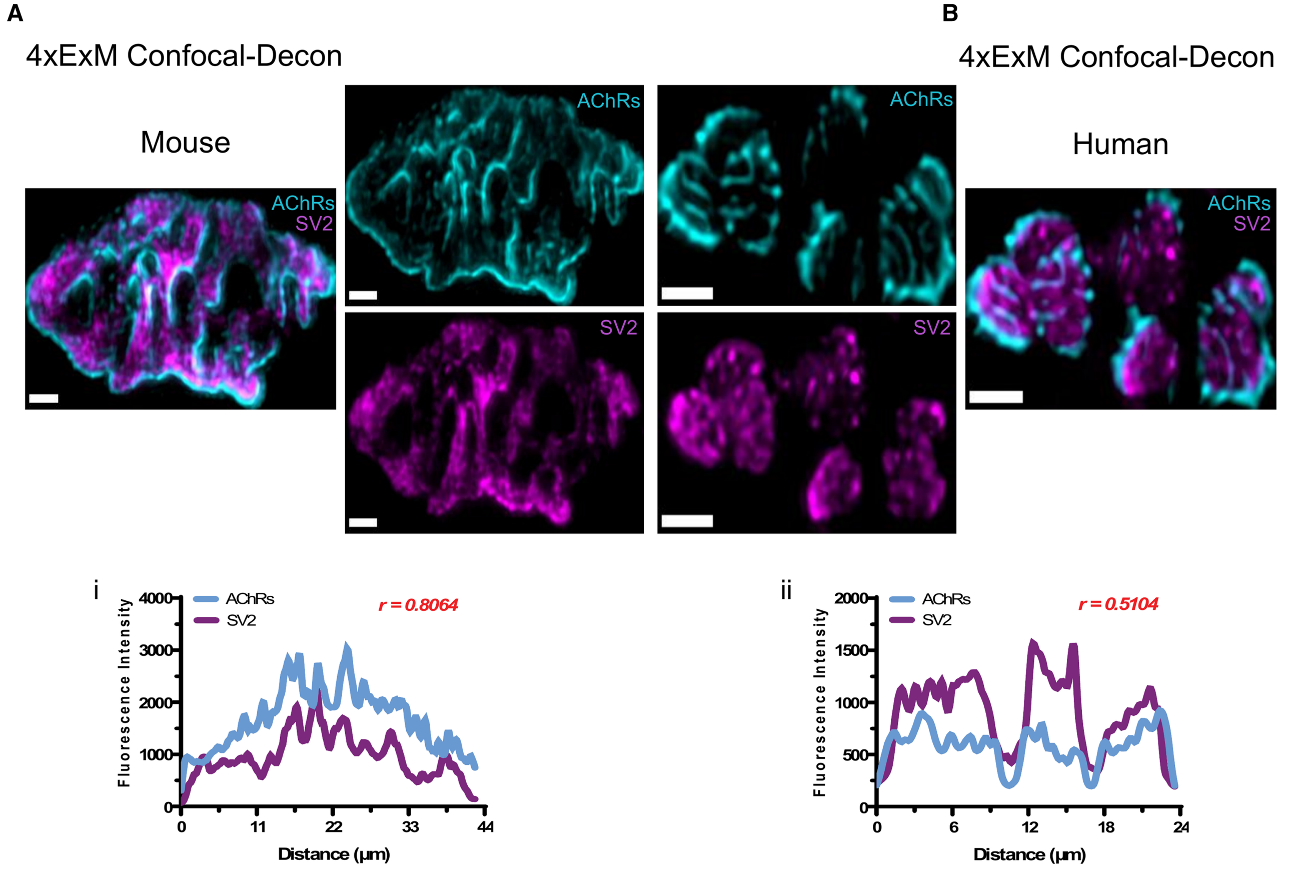

Figure 6. Differential alignment of pre- and postsynaptic structures at human and mouse NMJs revealed using ExM

Representative micrographs of ≈4×ExM NMJs revealing AChR distribution (cyan) versus SV2 distribution (magenta) in mouse (A) and human (B). At mouse NMJs, qualitative analysis of the distribution of the two markers suggested a homogeneous distribution of SV2 (e.g., synaptic vesicles) over the entire region of AChRs at the endplate, which was supported by quantitative analysis of the fluorescence intensity profiles (Pearson correlation of 0.8064) (i). In contrast, discernible puncta of SV2 labeling were evident at human NMJs, resulting in a greater spatial dissociation between the SV2 and AChR signals in the fluorescence intensity profiles (Pearson correlation of 0.5104) (ii). Scale bars: 4 μm.

(Figure 6Bii). This suggests that the localization of synaptic vesicle pools in human nerve terminals is more heterogeneous than that found in mouse nerve terminals. This finding is consistent with previous reports suggesting that active zones have a more punctate distribution in human NMJs.[7] Importantly, it should be noted that the fluorescence intensity profiles obtained from raw confocal images of unexpanded NMJs do not offer sufficient resolution to reveal these potentially important differences between the two species, even when using high-magnification objective lenses (Figure S5).

Finally, we used comparative ExM imaging of NMJs from mice and humans to address NaV1.4 distribution at the NMJ. NaV1.4 channels are known to be highly concentrated in the depths of the junctional folds of the motor endplate, while AChRs are predominantly localized at the upper crests of these folds. As such, it is unsurprising that ExM images of both mouse (Figure 7A) and human (Figure 7B) NMJs revealed a reciprocal pattern of localization between AChRs and NaV1.4 channels. At the human NMJ, however, NaV1.4 labeling extended much further beyond the territory occupied by AChRs than in mice, leading to a more pronounced NaV1.4 "rim" in human NMJs. In addition, there was a greater discrepancy between the fluorescence intensity profiles in human NMJs ($r = 0.65$) (Figure 7Bii) compared with mouse NMJs ($r = 0.85$) (Figure 7Ai). Given the fact that NaV1.4 channels are located in the depth of the junctional folds, these data support the suggestion that endplates at human NMJs are distinguished by more extensive postsynaptic folding, a characteristic that may compensate for the overall small size of human NMJs by magnifying neurotransmission.[2]

The fluorescent intensity profile analyses were conducted across the entire NMJ (excluding black areas with no signal). To clarify this approach, we included an explanatory figure (Figure S6) illustrating the method used to select the fluorescent intensity profile. This analysis approach enables the assessment of intensity profiles throughout the entire NMJ, providing insights into the spatial distribution of AChRs, SV2, and NaV1.4 within the NMJ. We propose that this method offers a more accurate representation of the spatial distribution of these targets compared

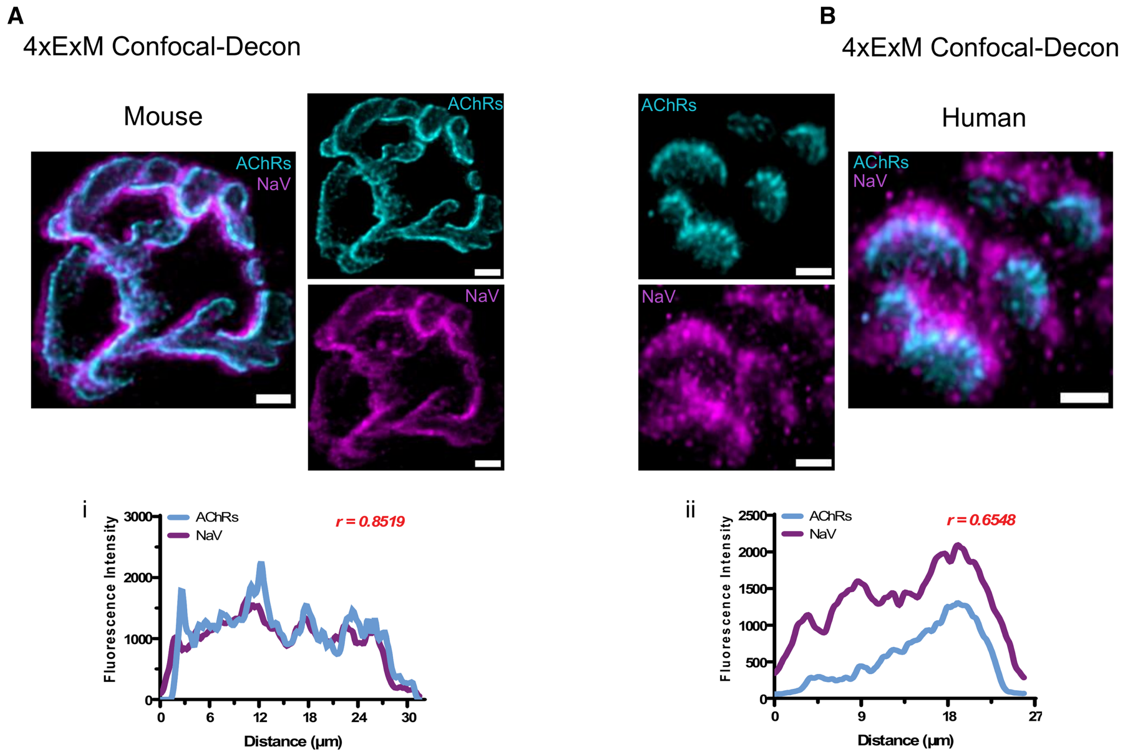

Figure 7. Differential alignment of NaV1.4 channels and AChRs at human and mouse NMJs revealed using ExM

Representative micrographs of ≈4×ExM NMJs revealing AChR distribution (cyan) versus NaV1.4 distribution (magenta) in mouse (A) and human (B). At mouse NMJs, qualitative analysis of the distribution of the two markers suggested a close matching of AChRs and NaV1.4 channels, which was supported by quantitative analysis of the fluorescence intensity profiles (Pearson correlation of 0.8519) (i). In contrast, NaV1.4 labeling was observed to extend well beyond the AChR domain at human NMJs, resulting in a greater spatial dissociation between the NaV1.4 and AChR signals in the fluorescence intensity profiles (Pearson correlation of 0.6548) (ii). Scale bars: 4 μm.

to the conventional approach of selecting a random line for analysis.

DISCUSSION

Currently, there is a paucity of literature concerning the nano-scale structure of human NMJs, largely due to the difficulties associated with applying SR imaging approaches to rare and hard-to-handle muscle biopsy samples. Here, we report on the adaptation of ExM as a technique suitable for use with human tissue samples[25] to elucidate nano-scale details of human NMJs. ExM offers a novel and accessible alternative approach to SR-based imaging of the NMJ, providing nano-scale insights into the form and function of the NMJ while overcoming the limitations of SR, including the high cost, need for specialized equipment, restricted fluorophore options, slow imaging speeds, and operational complexity. We propose that such an approach offers the opportunity to open up a new era of NMJ research, generating high-resolution insights into NMJ form and function in health and during disease.

Vertebrate NMJs possess distinctive structural features that are unique to each species,[26,27] making accurate characterization of these differences critical to the interpretation of data concerning NMJ form and function, as well as translational studies using animal-based disease models. For example, previous studies of the NMJ, utilizing both traditional light and electron microscopy (EM) approaches, have revealed that AChRs are positioned along the crests and upper parts of the junctional folds of the motor endplate but are absent from the lower regions.[28] The configuration of the folds and the selective organization of essential postsynaptic proteins within them serve to boost the amplification of synaptic currents, facilitating effective neurotransmission.[2] Beyond this basic arrangement, however, there is more limited knowledge about the precise distribution of the AChRs and junctional folds in individual endplate stripes, especially at the human NMJ. Here, we have used ExM to demonstrate clear differences in the spatial organization of the individual AChR stripes that comprise the motor endplate in human and mouse NMJs, with the human NMJ demonstrating larger stripes with wider spacing.

Using α-bungarotoxin to label AChRs, this striped pattern manifests as fluorescently intense bands juxtaposed between adjoining regions of diminished fluorescence. Since AChRs are known to aggregate predominantly at the crests of junctional folds while being almost entirely absent from the troughs, the conspicuous fluorescence observed in AChR stripes is likely to correspond to the peaks of these folds, which are demarcated

by regions devoid of AChR fluorescence within the infolded parts of the postsynaptic membrane. Furthermore, the large size of the AChR stripes at the human NMJ demonstrated here is likely to account for the spiculated and otherwise unusual appearances of the motor endplate noted in certain studies.[29] These observations support previous descriptions of the AChR distribution as stripes, bands, or "slits" noted in scanning EM[29–31] and more recent SR 3D-STORM and 3D-SIM studies.[12] Taken together, these results suggest distinct influences of the molecular and/or biophysical mechanisms regulating endplate morphology between humans and mice.

ExM revealed further insights that were not readily apparent when using conventional confocal microscopy. While presynaptic SV2 labeling still demonstrates a homogeneous distribution at the mouse NMJ upon ExM, the fluorescence intensity profile at the human NMJ demonstrated a punctate pattern, with visible hotspots of SV2 labeling on the expanded (post-ExM) images that were not visible on the unexpanded (pre-ExM/raw confocal) images, even when employing high-magnification objectives ($\times$60). These differences in the distribution of SV2 at human and mouse NMJs resemble previous findings in relation to 3D-STORM imaging of SNAP25 (an active zone protein), which was also aggregated into hotspots at the human NMJ.[7]

On the postsynaptic side of the NMJ, the bottom trough of each junctional fold is known to be rich in NaV1.4 channels.[2] Previous SR studies[12] have shown a high degree of overlap between AChRs and Na channels at the mouse NMJ, which we confirmed in the present study using ExM. In contrast, although human NaV1.4 channels demonstrated the same basic distribution, they were found to extend as a rim that protruded beyond the corresponding AChR area much more prominently than in mouse NMJs. This observation is in keeping with the fact that human junctional folds (which create the secondary synaptic clefts) are known to be much deeper than those of mouse NMJs.[32] Beyond these current observations, however, the existing literature is otherwise scant in relation to the distribution of NaV1.4 channels at human NMJs, and further studies will be required to explore its implication with regard to disease and aging.

In conclusion, we have demonstrated the feasibility of ExM as a powerful technique for comparative analysis of NMJ architecture, complementing existing SR approaches and making nanoscale imaging more widely available to the research community. We detail a robust and reproducible ExM protocol that has enabled us to overcome common challenges inherent in ExM, including gel drifting during imaging, dim fluorescent signals, and other difficulties associated with the use of high-magnification objectives (short working distance) in thick tissues.[18] In comparison with other, more established SR techniques, ExM is a relatively straightforward and cost-effective method for the generation of high-resolution images of similar quality.[14]

Limitations of the study

Although we demonstrate the successful development and application of ExM to the study of human NMJs, the quality of the original source material remains a critical determinant of success. Previous and current attempts to immunohistochemically label NMJs in human muscle tissue obtained postmortem proved unsuccessful, meaning that fresh biopsy samples are required. This limits the availability of suitable human experimental tissue available for such studies. Moreover, biopsy protocols often require strong collaborative links with clinical teams as well as robust ethical oversight and approvals.

Even when suitable experimental tissue samples can be obtained, the yield—with regard to the number of NMJs imaged per sample—remains low when applying ExM. This is impacted by several steps of the protocol, including the need to start with a small, teased tissue sample, as well as issues surrounding the imaging of NMJs positioned deeper within the sample. As a result, a larger number of tissue samples are often required for robust quantitative assessment of NMJ nano-structure when using ExM compared to more standard (albeit lower-resolution) techniques.

RESOURCE AVAILABILITY

Lead contact

Further information and requests for resources and reagents should be directed to and will be fulfilled by the lead contact, Thomas H. Gillingwater, via t.gillingwater@ed.ac.uk.

Materials availability

This study did not generate new unique reagents.

Data and code availability

- The data supporting the findings of this study are available from the lead contact/corresponding author upon request.
- This paper does not report original code.
- Any additional information required to reanalyze the data reported in this paper is available from the lead contact/corresponding author upon request.

ACKNOWLEDGMENTS

This work was supported by small project funding from Anatomy@Edinburgh, University of Edinburgh (to T.H.G. and R.A.J.); a PhD studentship (to A.R. and T.H.G.) from King Saud bin Abdulaziz University for Health Sciences, through the Saudi Cultural Bureau, London; and a UK Research and Innovation grant awarded to I.J. (MR/S03241X/1). The authors would like to thank the nursing staff in the theaters at St John's Hospital, Livingston, for assistance with tissue collection.

AUTHOR CONTRIBUTIONS

Conceptualization, A.R., T.M.D.S., I.J., and T.H.G.; formal analysis, A.R.; investigation, A.R.; methodology, A.R., T.M.D.S., A.A. (assisted), and P.A.R.; validation, A.R., T.M.D.S., R.A.J., I.J., and T.H.G.; visualization, A.R.; writing – original draft, A.R.; writing – review & editing, A.R., T.M.D.S., P.A.R., R.A.J., I.J., and T.H.G.; clinical coordination, P.A.R.; ethics coordination, R.A.J.; supervision, T.H.G.

DECLARATION OF INTERESTS

The authors declare no competing interests.

STAR★METHODS

Detailed methods are provided in the online version of this paper and include the following:

- KEY RESOURCES TABLE
- EXPERIMENTAL MODEL AND STUDY PARTICIPANT DETAILS
  - (Human subjects) muscle biopsy collection and dissection
  - (Animal tissues) muscle collection and dissection

CellPress
OPEN ACCESS

- METHOD DETAILS
  - Microdissection
  - NMJ immunohistochemistry
  - ExM protocol
  - Imaging
- QUANTIFICATION AND STATISTICAL ANALYSIS
  - Image analysis
  - Statistical analysis

SUPPLEMENTAL INFORMATION

Supplemental information can be found online at https://doi.org/10.1016/j.crmeth.2025.101082.

Received: October 8, 2024
Revised: February 19, 2025
Accepted: May 20, 2025
Published: June 16, 2025

REFERENCES

1. Iyer, S.R., Shah, S.B., and Lovering, R.M. (2021). The Neuromuscular Junction: Roles in Aging and Neuromuscular Disease. Int. J. Mol. Sci. *22*, 8058. https://doi.org/10.3390/ijms22158058.
2. Slater, C.R. (2017). The Structure of Human Neuromuscular Junctions: Some Unanswered Molecular Questions. Int. J. Mol. Sci. *18*, 2183. https://doi.org/10.3390/ijms18102183.
3. Rudolf, R., Khan, M.M., Labeit, S., and Deschenes, M.R. (2014). Degeneration of neuromuscular junction in age and dystrophy. Front. Aging Neurosci. *6*, 99. https://doi.org/10.3389/fnagi.2014.00099.
4. Huijbers, M.G., Marx, A., Plomp, J.J., Le Panse, R., and Phillips, W.D. (2022). Advances in the understanding of disease mechanisms of autoimmune neuromuscular junction disorders. Lancet Neurol. *21*, 163–175. https://doi.org/10.1016/S1474-4422(21)00357-4.
5. Alhindi, A., Boehm, I., and Chaytow, H. (2022). Small junction, big problems: Neuromuscular junction pathology in mouse models of amyotrophic lateral sclerosis (ALS). J. Anat. *241*, 1089–1107. https://doi.org/10.1111/joa.13463.
6. Li, L., Xiong, W.C., and Mei, L. (2018). Neuromuscular Junction Formation, Aging, and Disorders. Annu. Rev. Physiol. *80*, 159–188. https://doi.org/10.1146/annurev-physiol-022516-034255.
7. Jones, R.A., Harrison, C., Eaton, S.L., Llavero Hurtado, M., Graham, L.C., Alkhammash, L., Oladiran, O.A., Gale, A., Lamont, D.J., Simpson, H., et al. (2017). Cellular and Molecular Anatomy of the Human Neuromuscular Junction. Cell Rep. *21*, 2348–2356. https://doi.org/10.1016/j.celrep.2017.11.008.
8. Svoboda, K., and Yasuda, R. (2006). Principles of two-photon excitation microscopy and its applications to neuroscience. Neuron *50*, 823–839. https://doi.org/10.1016/j.neuron.2006.05.019.
9. Huang, B., Babcock, H., and Zhuang, X. (2010). Breaking the diffraction barrier: super-resolution imaging of cells. Cell *143*, 1047–1058. https://doi.org/10.1016/j.cell.2010.12.002.
10. Dani, A., Huang, B., Bergan, J., Dulac, C., and Zhuang, X. (2010). Super-resolution imaging of chemical synapses in the brain. Neuron *68*, 843–856. https://doi.org/10.1016/j.neuron.2010.11.021.
11. Nishimune, H. (2012). Molecular mechanism of active zone organization at vertebrate neuromuscular junctions. Mol. Neurobiol. *45*, 1–16. https://doi.org/10.1007/s12035-011-8216-y.
12. York, A.L., and Zheng, J.Q. (2017). Super-Resolution Microscopy Reveals a Nanoscale Organization of Acetylcholine Receptors for Trans-Synaptic Alignment at Neuromuscular Synapses. eNeuro *4*, ENEURO.0232-17.2017. https://doi.org/10.1523/ENEURO.0232-17.2017.
13. Gallagher, B.R., and Zhao, Y. (2021). Expansion microscopy: A powerful nanoscale imaging tool for neuroscientists. Neurobiol. Dis. *154*, 105362. https://doi.org/10.1016/j.nbd.2021.105362.
14. Zhuang, Y., and Shi, X. (2023). Expansion microscopy: A chemical approach for super-resolution microscopy. Curr. Opin. Struct. Biol. *81*, 102614. https://doi.org/10.1016/j.sbi.2023.102614.
15. Chen, F., Tillberg, P.W., and Boyden, E.S. (2015). Optical imaging. Expansion microscopy. Science *347*, 543–548. https://doi.org/10.1126/science.1260088.
16. Hümpfer, N., Thielhorn, R., and Ewers, H. (2024). Expanding boundaries - a cell biologist's guide to expansion microscopy. J. Cell Sci. *137*, jcs260765. https://doi.org/10.1242/jcs.260765.
17. Sheard, T.M.D., Hurley, M.E., Smith, A.J., Colyer, J., White, E., and Jayasinghe, I. (2022). Three-dimensional visualization of the cardiac ryanodine receptor clusters and the molecular-scale fraying of dyads. Philos. Trans. R. Soc. Lond. B Biol. Sci. *377*, 20210316. https://doi.org/10.1098/rstb.2021.0316.
18. Asano, S.M., Gao, R., Wassie, A.T., Tillberg, P.W., Chen, F., and Boyden, E.S. (2018). Expansion Microscopy: Protocols for Imaging Proteins and RNA in Cells and Tissues. Curr. Protoc. Cell Biol. *80*, e56. https://doi.org/10.1002/cpcb.56.
19. Wassie, A.T., Zhao, Y., and Boyden, E.S. (2019). Expansion microscopy: principles and uses in biological research. Nat. Methods *16*, 33–41. https://doi.org/10.1038/s41592-018-0219-4.
20. Gaudreau-Lapierre, A., Mulatz, K., Béïque, J.C., and Trinkle-Mulcahy, L. (2021). Expansion microscopy-based imaging of nuclear structures in cultured cells. STAR Protoc. *2*, 100630. https://doi.org/10.1016/j.xpro.2021.100630.
21. Zhu, C., Wang, A., Chen, L., Guo, L., Ye, J., Chen, Q., Wang, Q., Yao, G., Xia, Q., Cai, T., et al. (2021). Measurement of expansion factor and distortion for expansion microscopy using isolated renal glomeruli as landmarks. J. Biophot. *14*, e202100001. https://doi.org/10.1002/jbio.202100001.
22. Pedersen, T.H., Macdonald, W.A., Broch-Lips, M., Halldorsdottir, O., and Baekgaard Nielsen, O. (2021). Chloride channel inhibition improves neuromuscular function under conditions mimicking neuromuscular disorders. Acta Physiol. *233*, e13690. https://doi.org/10.1111/apha.13690.
23. Nishimune, H., Badawi, Y., Mori, S., and Shigemoto, K. (2016). Dual-color STED microscopy reveals a sandwich structure of Bassoon and Piccolo in active zones of adult and aged mice. Sci. Rep. *6*, 27935. https://doi.org/10.1038/srep27935.
24. Ackermann, F., Waites, C.L., and Garner, C.C. (2015). Presynaptic active zones in invertebrates and vertebrates. EMBO Rep. *16*, 923–938. https://doi.org/10.15252/embr.201540434.
25. Klimas, A., Gallagher, B.R., Wijesekara, P., Fekir, S., DiBernardo, E.F., Cheng, Z., Stolz, D.B., Cambi, F., Watkins, S.C., Brody, S.L., et al. (2023). Magnify is a universal molecular anchoring strategy for expansion microscopy. Nat. Biotechnol. *41*, 858–869. https://doi.org/10.1038/s41587-022-01546-1.
26. Boehm, I., Alhindi, A., Leite, A.S., Logie, C., Gibbs, A., Murray, O., Farrukh, R., Pirie, R., Proudfoot, C., Clutton, R., et al. (2020). Comparative anatomy of the mammalian neuromuscular junction. J. Anat. *237*, 827–836. https://doi.org/10.1111/joa.13260.
27. Zou, S., and Pan, B.X. (2022). Post-synaptic specialization of the neuromuscular junction: junctional folds formation, function, and disorders. Cell Biosci. *12*, 93. https://doi.org/10.1186/s13578-022-00829-z.
28. Fertuck, H.C., and Salpeter, M.M. (1974). Localization of acetylcholine receptor by 125I-labeled alpha-bungarotoxin binding at mouse motor endplates. Proc. Natl. Acad. Sci. USA *71*, 1376–1378. https://doi.org/10.1073/pnas.71.4.1376.
29. Desaki, J., and Uehara, Y. (1987). Formation and maturation of subneural apparatuses at neuromuscular junctions in postnatal rats: a scanning and

transmission electron microscopical study. Dev. Biol. *119*, 390–401. https://doi.org/10.1016/0012-1606(87)90044-3.

30. Ogata, T., and Yamasaki, Y. (1985). The three-dimensional structure of motor endplates in different fiber types of rat intercostal muscle. A scanning electron-microscopic study. Cell Tissue Res. *241*, 465–472. https://doi.org/10.1007/BF00214564.

31. Fahim, M.A., Holley, J.A., and Robbins, N. (1984). Topographic comparison of neuromuscular junctions in mouse slow and fast twitch muscles. Neuroscience *13*, 227–235. https://doi.org/10.1016/0306-4522(84)90273-2.

32. Badawi, Y., and Nishimune, H. (2020). Impairment Mechanisms and Intervention Approaches for Aged Human Neuromuscular Junctions. Front. Mol. Neurosci. *13*, 568426. https://doi.org/10.3389/fnmol.2020.568426.

33. Alhindi, A., Boehm, I., Forsythe, R.O., Miller, J., Skipworth, R.J.E., Simpson, H., Jones, R.A., and Gillingwater, T.H. (2021). Terminal Schwann cells at the human neuromuscular junction. Brain Commun. *3*, fcab081. https://doi.org/10.1093/braincomms/fcab081.

CellPress
OPEN ACCESS

STAR★METHODS

KEY RESOURCES TABLE

REAGENT or RESOURCE	SOURCE	IDENTIFIER
Antibodies		
Mouse anti-SV2 IgG	Developmental Studies Hybridoma Bank	Cat#2315387; RRID: AB_2315387
Mouse anti-NaV1.4 N255/38 antibody	Antibodies Incorporated	Cat#N255/38; RRID: AB_2877201
Alexa 594-goat anti-mouse IgG	Thermo Fisher Scientific	Cat#A-11005; RRID: AB_2534073
Alexa 488-α-bungarotoxin	Thermo Fisher Scientific	Cat#B13422
Biological samples		
Human dorsal interosseous muscle biopsies	This study	N/A
Chemicals, peptides, and recombinant proteins		
Paraformaldehyde (PFA)	Electron Microscopy Sciences	Cat#15710
Bovine Serum Albumin (BSA)	VWR International Radnor	Cat#97061-422
Sodium Acrylate (SA)	Sigma-Aldrich, UK	Cat#408220-25g
Acrylamide	Sigma-Aldrich, UK	Cat#A9099-25g
N, N-methylenebisacrylamide	Sigma-Aldrich, UK	Cat#274135
Sodium Chloride (NaCl)	Sigma-Aldrich, UK	Cat#S7653-1kg
N, N, N′, N′-Tetramethylethylenediamine (TEMED)	Sigma-Aldrich, UK	Cat#T7024
Ammonium persulfate (APS)	Sigma-Aldrich, UK	Cat#A3678-25g
Tris	Thermo Fisher Scientific, UK	Cat#AM9855g
Ethylenediaminetetraacetic acid (EDTA)	Sigma-Aldrich, UK	Cat#EDS-100g
Guanidine Hydrochloride	Sigma-Aldrich, UK	Cat#G327225g
Triton X-100	Sigma-Aldrich, UK	Cat#T9284
Acryloyl-X (AcX)	Thermo Fisher Scientific, UK	A20770 5mg
DMSO	Biotium, USA	90082-BT
Poly-L-lysine	Sigma-Aldrich, UK	P8920-100mL
Phosphate buffered saline (PBS)	This study	NA
Proteinase K	New England Biolabs, UK	P8107S 2mL
Experimental models: Organisms/strains		
FVB mice	Biological Research Resources – University of Edinburgh	N/A
Software and algorithms		
Hugyens software: Classic Maximum Likelihood Estimation (CMLE)	Scientific Volume Imaging (SVI)	https://svi.nl/Huygens-Confocal-Software
Fiji software	FIJI	https://imagej.net/Fiji
GraphPad Prism software	GraphPad Software, USA	Version 10

EXPERIMENTAL MODEL AND STUDY PARTICIPANT DETAILS

(Human subjects) muscle biopsy collection and dissection

Four human Dorsal Interosseous (*DIO)* muscle specimens were obtained via biopsies (where possible spanning from the origin to the insertion) from three anonymous middle-aged patients of both sexes who were undergoing hand surgery procedures for unrelated clinical reasons (e.g., reconstructive fracture surgeries). All human work was approved by the NHS Grampian Ethics Committee (REC ref. 20/NS/0008; Protocol number: AC18077; IRAS project ID: 244717). Samples were fixed immediately once harvested for 1 h in 4% of Paraformaldehyde (PFA).

(Animal tissues) muscle collection and dissection

Since human biopsies were harvested from *DIO*, we maintained comparability by dissecting out forelimb interosseous muscles from four adult (~12 weeks old) mice, encompassing both sexes and both sides. Briefly, mice were euthanized using a Schedule 1

procedure approved by the UK Home Office. Forelimbs were cut at the elbow joints, and the skin along with the underlying fascia was removed using fine forceps and scissors. Distally, on the dorsal side, pins were used to secure the forepaws on a 90 mm Petri dish under the dissection microscope. After identifying the interosseous muscles, the metacarpal bones were carefully dislocated from the carpal bones and completely removed using curved forceps. Connective tissue was then detached from and between the muscle fibers. Muscle specimens were fixed immediately once harvested for 1 h in 4% (v/v) PFA in 1x PBS.

METHOD DETAILS

Microdissection

Muscle fiber teasing was carried out after three washes in 1x Phosphate buffered saline (PBS).[7,33] Briefly, small groups of muscle fibers spanning from the origin to the insertion were dissected out from the biopsy sample. These were then subsequently microdissected under a dissection microscope into small fiber bundles, each comprising less than 10 individual fibers (acquiring a small sample is crucial to allow proper digestion and even expansion eventually). To ensure minimal background staining, enhance antibody penetration and allow an even maximum expansion, residual fat and connective tissue were also removed via microdissection. Typically, following the microdissection process, each specimen yielded a few muscle bundles, each consisting of ~4–8 individual muscle fibers. Each bundle was subsequently used to create a single ExM gel, typically containing 4–8 NMJs.

NMJ immunohistochemistry

Following microdissection, muscle fibers were transferred in 24-well plates and subjected to the subsequent immunolabelling protocol (Step 1 in Video S1). Subsequent permeabilisation with Triton X-(4%) was applied to facilitate antibody penetration, whilst a blocking solution consisting of 4% of Bovine serum albumin (BSA) (w/v) and 2% (v/v) Triton X-(Sigma-Aldrich, UK) was applied to prevent non-specific binding. Muscle bundles were then subjected to overnight incubation at 4°C with a primary antibody, either mouse anti-SV2 IgG (Developmental Studies Hybridoma Bank, University of Iowa) or mouse anti-NaV1.4 IgG (Anti-Nav1.4 Sodium Channel Antibody-N255/38 from Antibodies Incorporated), at dilutions of 1:50 and 1:500, respectively. Following incubation, the samples underwent four consecutive washes with 1% PBS, each lasting 20 min. Subsequently, Alexa 594-Goat anti-mouse IgG (Thermo Fisher Scientific, Cat number: A-11005), at a dilution of 1:400, was utilized as a secondary antibody and incubated for 5 h at room temperature. Alexa 488 α-Bungarotoxin (BTX, Alexa Fluor 488 conjugate, B13422, Invitrogen, Thermo Fisher Scientific) was added for 30 min at a dilution of 1:500 to label AChRs.

ExM protocol

On the same 24-well plates (Step 1 in Video S1), direct anchoring of fluorescent molecules in the labeled muscle sample was achieved by adding 0.1 mg/mL of anchoring reagent Acryloyl-X (AcX; Thermo Fisher Scientific, UK) from an AcX stock of 10 mg/mL in (DMSO; Biotium, USA) overnight at 4°C. Samples were then washed twice with 1x PBS before adding a monomer solution for polymerization for 2 h at 4°C. Next, the gel chamber was prepared, using a slide covered entirely with parafilm tape (Step 2 in Video S1) with small pieces of cut coverslips placed on top as walls or spacers (Step 3 in Video S1) and Figure S1A).

A stock of monomer solution was prepared in a 10 mL Falcon tube, containing the following components and concentrations: sodium acrylate (SA) (Sigma-Aldrich, UK) at 38 g/100 mL, acrylamide (Sigma-Aldrich, UK) at 50 g/mL, N,N-methylenebisacrylamide (Sigma-Aldrich, UK) at 2 g/mL, NaCl (Sigma-Aldrich, UK) at 29.2 g/mL, 10x PBS at 1 mL, and deionized water (dH2O) at 0.9 mL. The total volume of the prepared stock monomer solution was 9.4 mL. For each experiment, the final concentrations were extracted from the stock as follows: sodium acrylate at 8.6 g/2.5 mL, acrylamide at 2.5 g/2.5 mL, N,N-methylenebisacrylamide at 0.15 g/2.5 mL, NaCl at 11.7 g/2.5 mL, diluted using 1x PBS. Individual aliquots of the monomer solution were measured into an Eppendorf tube with a total volume of 300 μL. The solution can be stored at −20°C for a couple of months.

Polymerization of the gel (gelation) was achieved by sequentially applying a 300 μL gel solution containing 282 μL of monomer solution, 6 μL PBS, 6 μL 10% of ammonium persulfate (APS; Sigma-Aldrich, UK), and 6 μL of N,N,N′,N′-Tetramethylethylenediamine (TEMED; Sigma Aldrich, UK). The muscle sample was carefully placed onto a coated (250 μL of poly-L-lysine for 1 h) glass coverslip (18 × 18 mm, No 1.5) (Step 4 in Video S1) then gel solution (150 μL) was added to the chambers to create an individual expandable gel. The coverslip (where the muscle sample is attached) was then flipped onto the gel chamber (Step 5 in Video S1). Gelation was allowed to take place for 2 h in a 37°C incubator.

To detach the gel from the gelation chamber, the upper coverslip (where the muscle sample was affixed) was carefully removed using a blade (and remove the spacers). Typically, the gel adhered to the upper coverslip (Figure S1. B). The gel was trimmed using a blade (Step 6 in Video S1), with an asymmetric shape to facilitate subsequent orientation of the sample. At this time the gel pre-ExM dimensions were measured.

The coverslip was then transferred to a six-well plate (one gel per well), with the gel facing upwards (Step 7 in Video S1). The digestion step was achieved by adding the digestive buffer, which was prepared in a 200 mL beaker and contained the following components: 10x PBS, 50 mM Tris (Thermo Fisher Scientific, UK), 1 mM Ethylenediaminetetraacetic acid (EDTA-Sigma-Aldrich, UK), 0.8 M Guanidine Hydrochloride (Sigma-Aldrich, UK), and 0.5% Triton X-100. Deionized water was added to achieve a total volume of 100 mL. The buffer was then aliquoted into ten Falcon tubes, each containing 10 mL, and subsequently frozen. Immediately prior to the experiment, 100 μL of Proteinase K (New England Biolabs, UK) with a working concentration 8 units/ml (1:100 dilution) was

added to 10 mL of the digestive buffer. The six-well plate was then filled with approximately 3mL of the digestive mixture per gel. Digestion required 18–20 h of incubation at 37°C to allow for the digestion of muscle samples and subsequent even expansion of the gel (this is also required to prevent cracking and potential distortion).

For the expansion step, the gel was removed (still attached to the coverslip) from the digestion buffer and transferred onto a 150 mm Petri dish before four 30-min washes with ddH_2O in darkness (Step 8 in Video S1). It is important to ensure total immersion of the gel in the ddH_2O as well as complete darkness. Once the gel reached a plateau state and detached from the coverslip, gel dimensions were measured and compared to pre-expansion measurements to ensure a 3.5–4 times expansion. Gel excision and mounting were performed by carefully trimming, orientating, and transferring the expanded gel using coverslips, flat metal spatulas, and a paintbrush (Step 9 in Video S1). Finally, the gels were transferred to a customized acrylic imaging chamber with a square cut-out (Step 10 in Video S1), designed for attaching a #1.5 coated coverslip to minimize drift during imaging.

Imaging

A Nikon A1R FLIM confocal laser scanning microscope with 20x/0.75 NA air objective was used to capture 16-bit, 512 x 512-pixel frame size, Z—stack images with 0.5 μm interval, at 9x zoom; red channel—561 nm excitation; green channel—488 nm excitation. NMJs selected for imaging were oriented *en face* and were located close to the bottom surface of the gel (the imaging depth range is ≈ 40–60 μm). Gels were balanced with regards to hydration by adding water droplets or absorbing it with filter paper to prevent the potential collapse of the gel and/or drifting during imaging.

QUANTIFICATION AND STATISTICAL ANALYSIS

Image analysis

As an initial step, confocal z-stacks images were uploaded into Huygens software, where the deconvolution Classic Maximum Likelihood Estimation (CMLE) algorithm was automatically computed based on the Point Spread Function (PSF) of the microscope, which had been previously calculated using fluorescent microspheres. To mimic the experimental conditions of the gel and minimize refractive index discrepancy, fluorescent microsphere analysis was performed using a water immersion objective. Deconvolved z stack images were loaded into Fiji software and collapsed into maximum intensity projections for pre-processing and measurements. The width of AChR stripes was measured on an adjusted and binarized by thresholding image. Measurement of AChR stripe length and the distance between them was based on the skeletonization plugin (Figure S2). To determine the level of overlapping/colocalization between AChRs and SV2 or NaV1.4 signals, profile intensity for fluorescent analysis was used.

Statistical analysis

GraphPad Prism Software (Version 10) was used for all statistical analyses and for preparing graphs. Statistical analysis for species comparisons (Human versus Mouse) was performed by using an unpaired t-test (two-tailed) and Pearson's correlation coefficient. Individual statistical tests with details and significance levels are referred to in the relevant text sections and corresponding figure legends. $p < 0.05$ was considered to be statistically significant.

Cell Reports Methods, Volume *5*

Supplemental information

Expansion microscopy reveals nano-scale insights into the human neuromuscular junction

Abdullah Ramadan, Thomas M.D. Sheard, Abrar Alhindi, Philippa A. Rust, Ross A. Jones, Izzy Jayasinghe, and Thomas H. Gillingwater

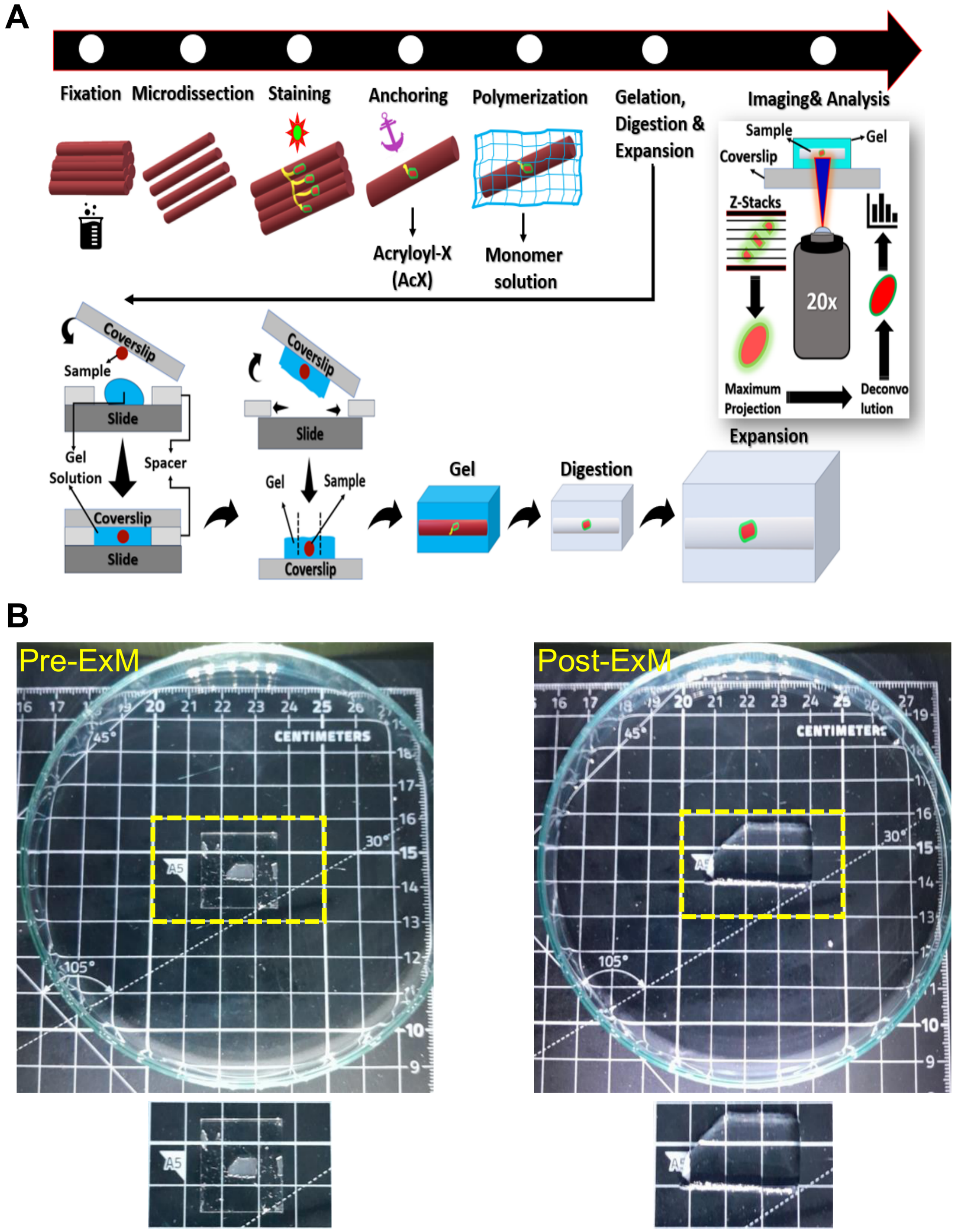

Figure S1: Expansion microscopy workflow and gel expansion in action, related to STAR Methods. (A) Schematic illustration of the workflow of the ExM NMJ protocol, which takes ~5 days to complete. At the beginning and after appropriate fixation of the muscle tissue with 4% PFA and fine dissection, samples are labelled/stained using conventional NMJ staining protocols (see Methods). Subsequently, samples are treated with Acryloyl-X SE (AcX) to anchor fluorescent proteins to the hydrogel before a monomer solution is applied to the sample. Following this, polymerization of the sample and formation of the hydrogel occur in the gel chamber between a coverslip (on top) and a slide (underneath). Digestion using proteinase K with digestive buffer is then applied to clear the specimen from any remaining tissue that would impede the even expansion of fluorescent proteins. Finally, samples can be expanded in ddH20 before imaging using a standard confocal microscope. **(B)** Representative photographs of the expansion process of a hydrogel loaded with muscle/NMJ samples. **(Left)** The gel pre-ExM on a petri dish. **(Right)** The gel expanded ≈4x. Note that the gel appears transparent and is evenly expanded with no signs of distortion. In this example the distances as follow: the X-axis in the pre-expansion status is 7.5 and after expansion is 30 mm. The pre-expansion distance of the Y-axis is 4.5 and 18 mm in post-expansion status.

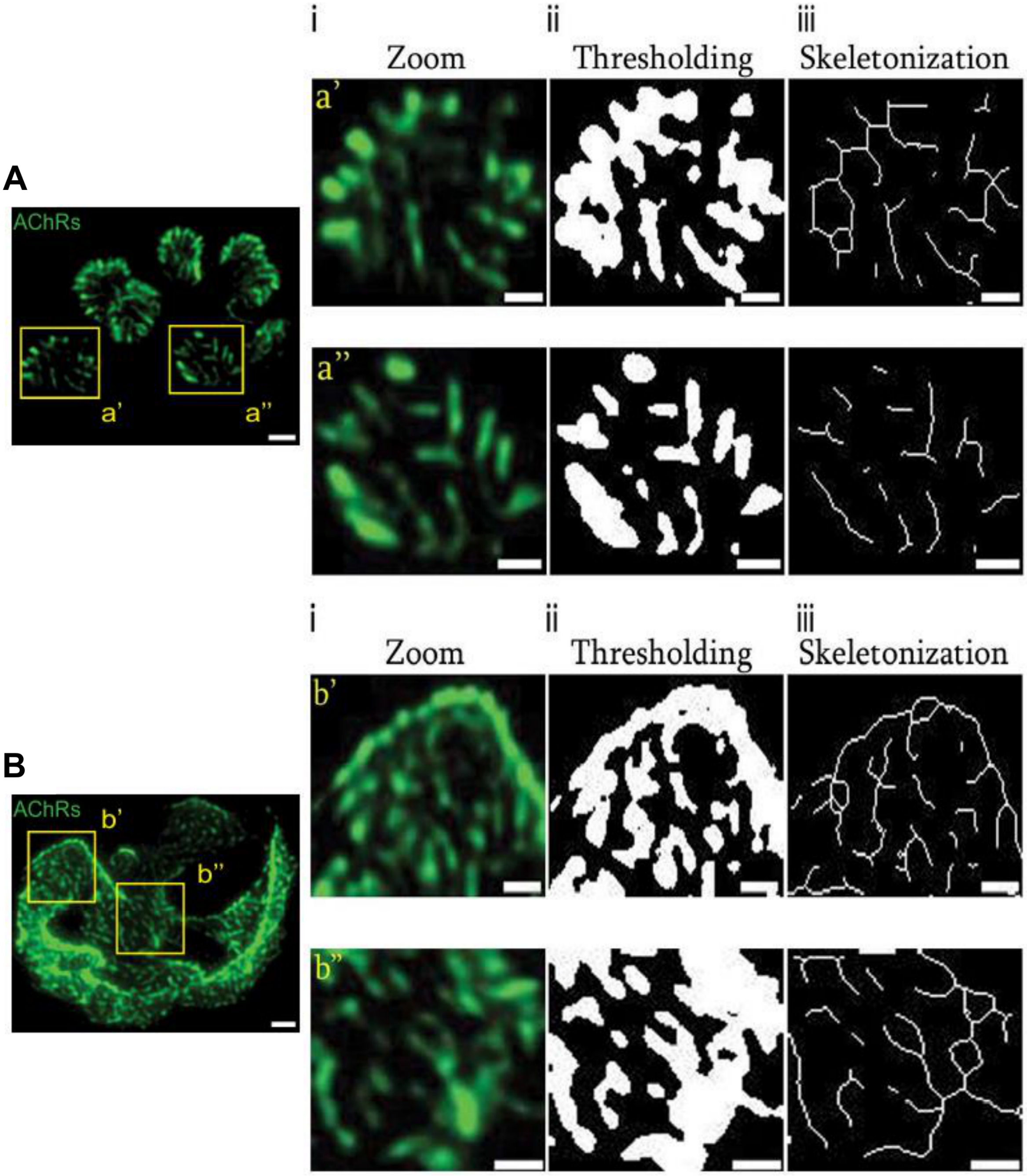

Figure S2: Image processing methodology for quantifying AChR distribution on ExM images, related to Figure 5. Example images of single synaptic boutons from a human NMJ **(A)** and an equivalent area from a mouse NMJ **(B)**. After zooming in **(i)**, measurement of AChR width was performed on thresholded images **(ii)**, whilst length and distance between stripes was measured using on skeletonization-processed images **(iii)**. Scale bars= 4μm corresponding to the pre-expansion dimension, while the zoomed-in images =2 μm pre-expansion dimension.

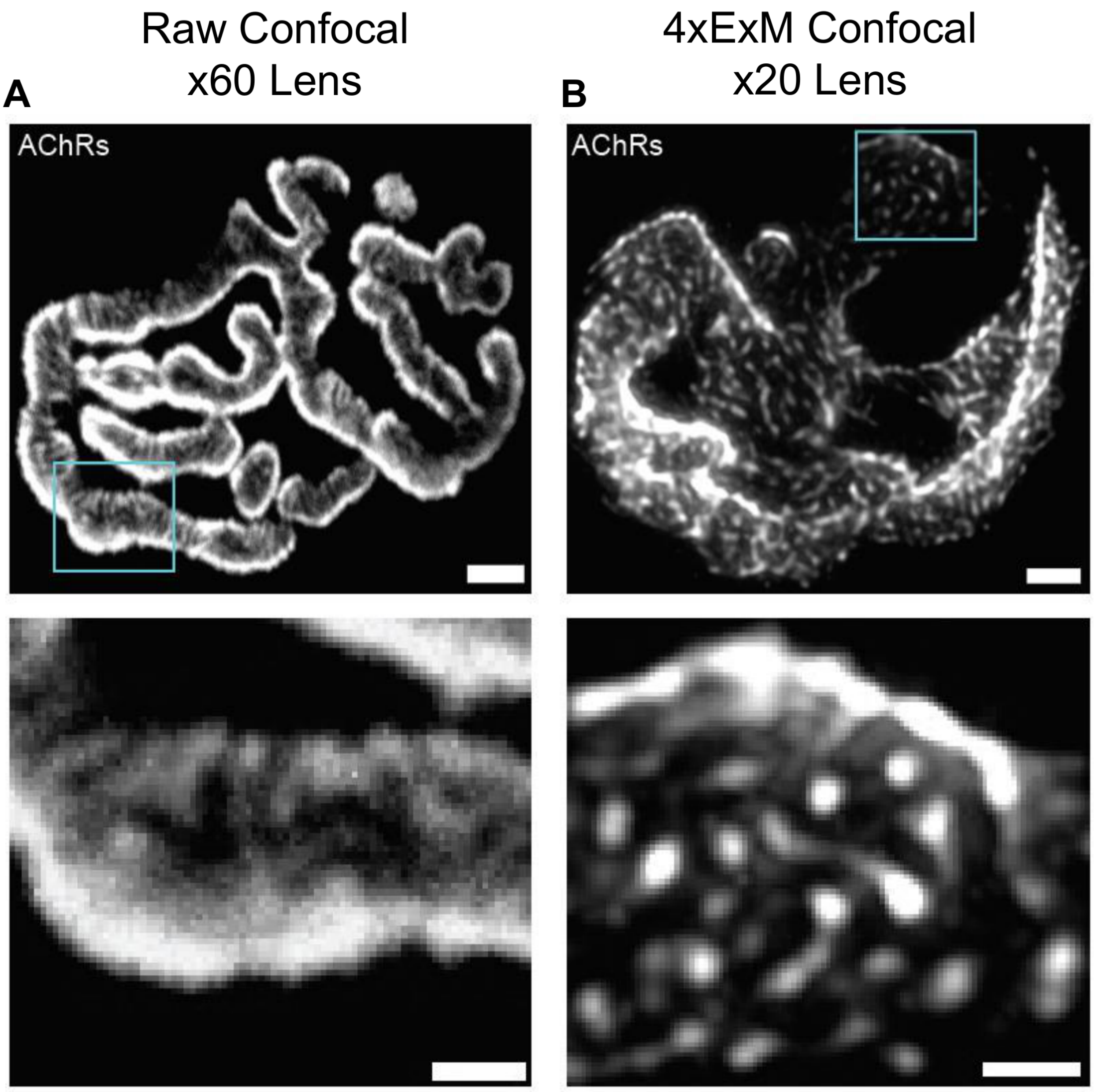

Figure S3: Increased resolution of mouse NMJ structure using ExM compared to standard confocal microscopy, related to Figure 3. Representative examples showing the superiority of 4xExM to standard confocal microscopy with respect to revealing NMJ ultrastructure in mouse tissues. **(A)** representative examples of raw confocal images of AChRs at a single mouse NMJ, acquired using a x60 objective, whereas **(B)** is an image of AChRs at an expanded (4x) NMJ captured using x20 objective. Scale bars =4 μm (in biological units =~1 μm pre-expansion). ExM images revealed significantly more details due to the increased spatial separation of fluorophores in the ExM protocol. When zooming in on a single bouton (bottom images), the expanded NMJs maintained good image quality and resolution, allowing for the identification and tracing of details that were not possible with raw confocal images, which lost resolution and became blurred upon zooming in. Scale bars in zoomed-in images =2 μm (in biological units = 500 nm).

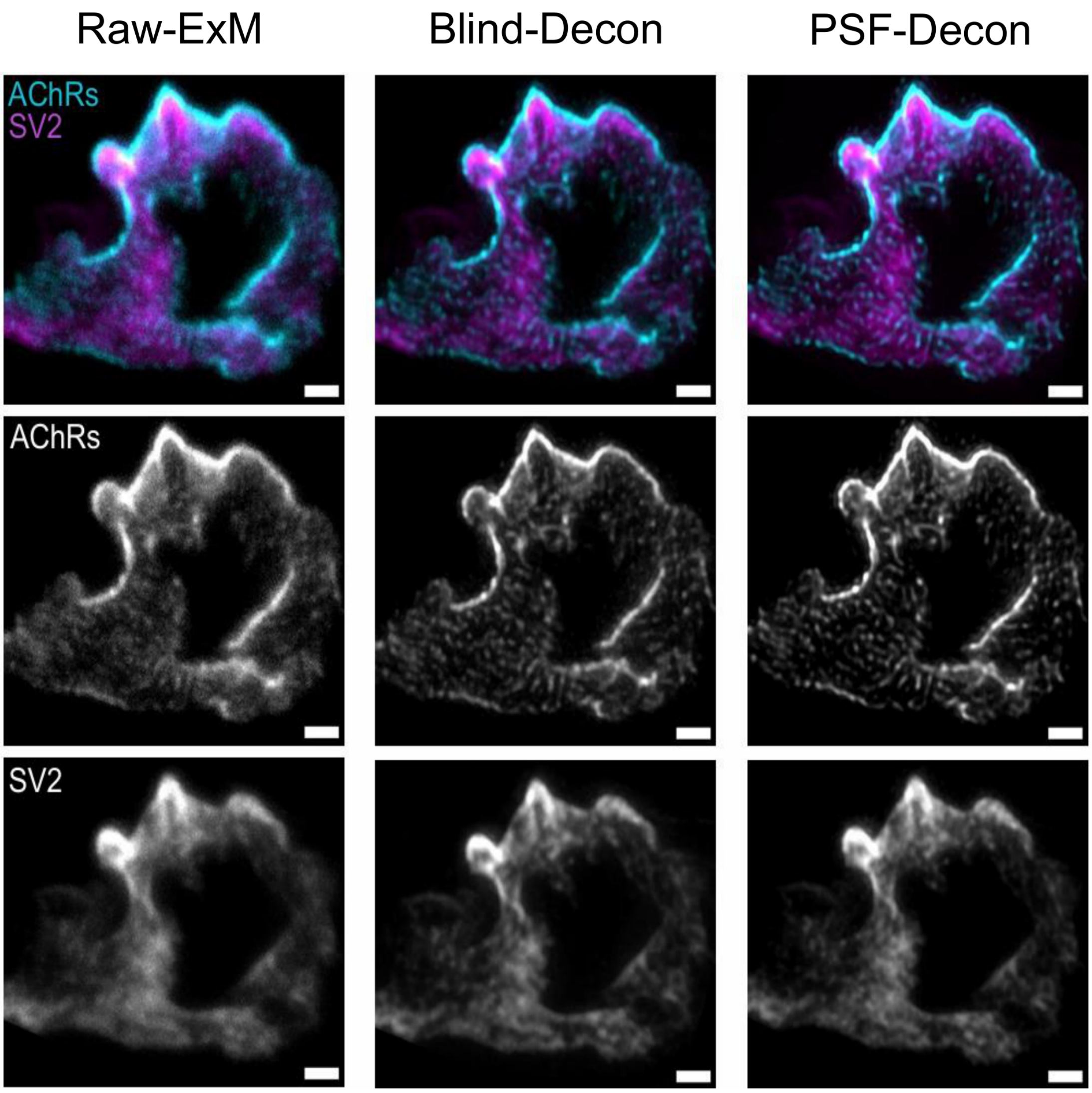

Figure S4: Comparison of different deconvolution techniques for use on ExM images, related to Figure 4. Series of images illustrating the effect of deconvolution on images of expanded (≈ x4) Mouse NMJs. Left panels show an unprocessed raw image, with middle columns showing the same images after applying the Classical Maximum Likelihood Estimation (CMLE) deconvolution algorithm based on the theoretical Point Spread Function (PSF), and the right columns showing the same images processed using an identical deconvolution algorithm but with the precise experimental PSF. NMJs were stained with α-Bungarotoxin for AChRs (in cyan) and SV2 for presynaptic vesicles (magenta). Notably, the deconvolution process led to a substantial enhancement in image contrast, noise reduction, and resolution in visualizing nano-scale structural details. Scale bars = 4 μm, (biological unit =1 μm pre-expansion).

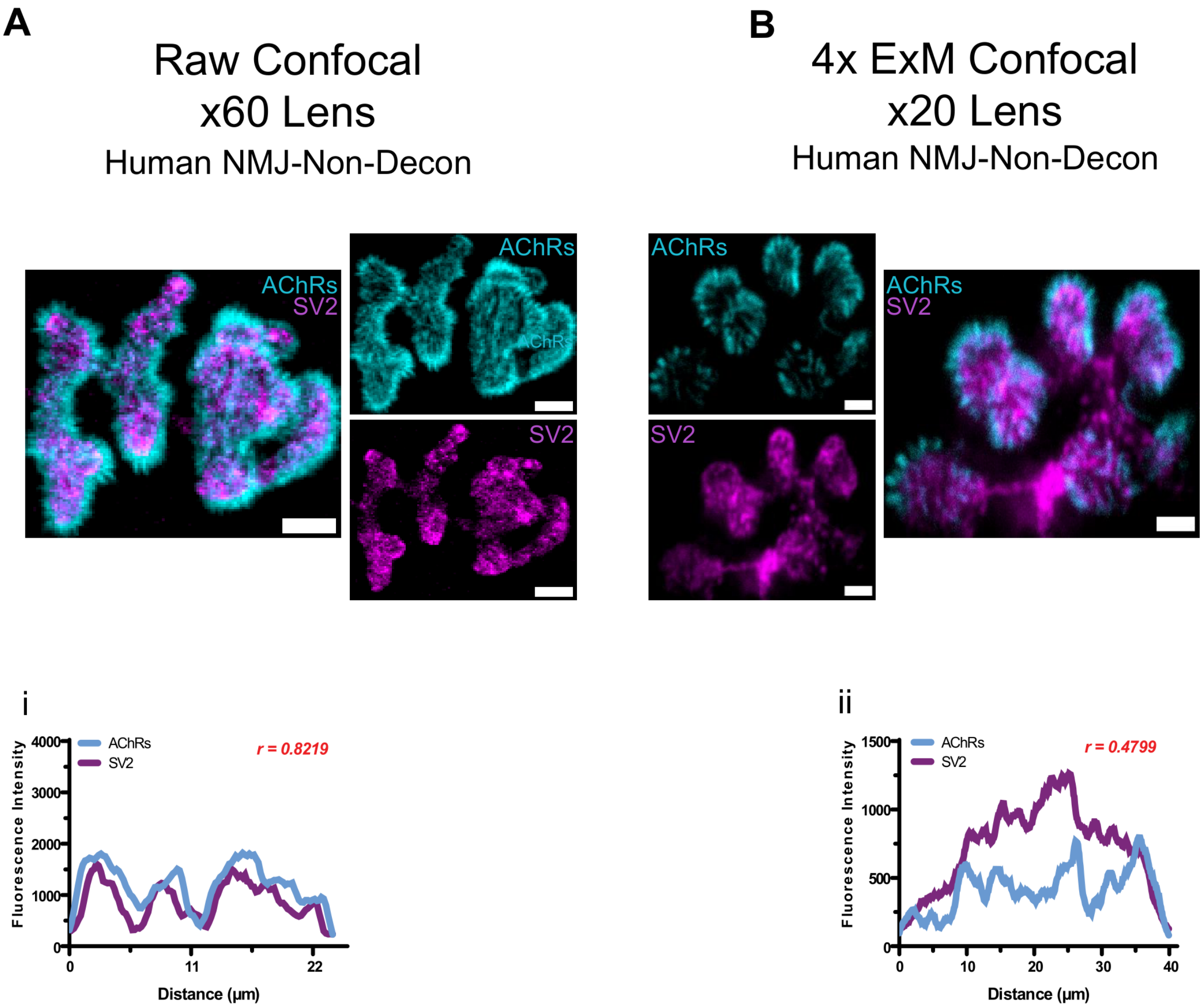

Figure S5: Differential alignment of pre-and post-synaptic structures at the human NMJ revealed by ExM is not detectable using standard confocal microscopy, related to Figure 6 and 7. Comparison of AChR (cyan) and SV2 (magenta) labelling at human NMJs imaged using **(A)** standard confocal microscopy pre-expansion or **(B)** 4x-ExM. Note that the punctate SV2 distribution was not as clear on the standard confocal micrographs. Quantitative analysis of fluorescence intensity profiles revealed no spatial separation in the standard preparations (Pearson correlation of 0.8219) **(i)**, in contrast to the greater spatial dissociation between SV2 and AChRs in the fluorescence intensity profiles from ExM images (Pearson correlation of 0.4799) **(ii)**. Scale bars = 4μm, corresponding to pre-expansion dimensions.

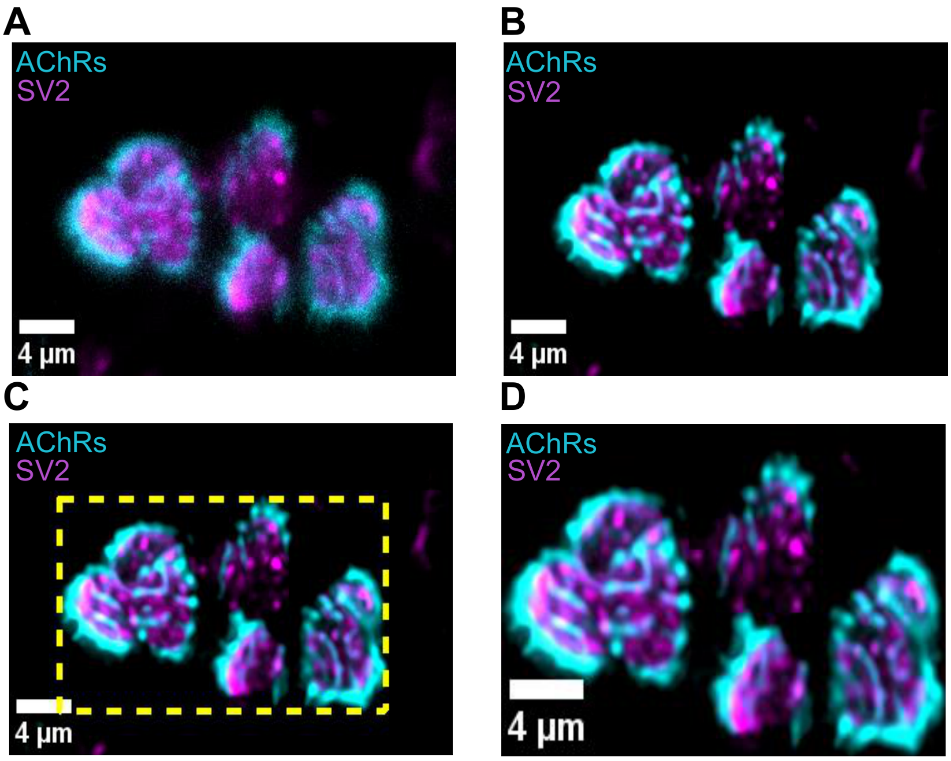

Figure S6: Fluorescence intensity profile selection example, related to Figure 6 and 7. Representative micrographs of ≈x4ExM NMJs revealing AChR distribution (cyan) versus SV2 distribution (magenta) in human NMJ. **(A)** shows the original confocal micrograph. **(B)** the same image after deconvolution. **(C)** The yellow-dotted quadrangle in **(B)** represents the selected region where the quantitative analysis profile was run. **(D)** the final cropped image as appears in the manuscript which reflects the entire NMJ, excluding the non-fluorescent area (black area) where the analysis was run and the Pearson correlation was calculated accordingly. Scale bars = 4µm.
